# Defence Responses of *Arabidopsis thaliana* to Infection by *Pseudomonas syringae* Are Regulated by the Circadian Clock

**DOI:** 10.1371/journal.pone.0026968

**Published:** 2011-10-31

**Authors:** Vaibhav Bhardwaj, Stuart Meier, Lindsay N. Petersen, Robert A. Ingle, Laura C. Roden

**Affiliations:** 1 Department of Molecular and Cell Biology, University of Cape Town, Rondebosch, South Africa; 2 Division of Chemistry, Life Science and Engineering, King Abdullah University of Science and Technology, Thuwal, Saudi Arabia; University of Massachusetts Amherst, United States of America

## Abstract

The circadian clock allows plants to anticipate predictable daily changes in abiotic stimuli, such as light; however, whether the clock similarly allows plants to anticipate interactions with other organisms is unknown. Here we show that *Arabidopsis thaliana* (Arabidopsis) has circadian clock-mediated variation in resistance to the virulent bacterial pathogen *Pseudomonas syringae* pv. *tomato* DC3000 (*Pst* DC3000), with plants being least susceptible to infection in the subjective morning. We suggest that the increased resistance to *Pst* DC3000 observed in the morning in Col-0 plants results from clock-mediated modulation of pathogen associated molecular pattern (PAMP)-triggered immunity. Analysis of publicly available microarray data revealed that a large number of Arabidopsis defence-related genes showed both diurnal- and circadian-regulation, including genes involved in the perception of the PAMP flagellin which exhibit a peak in expression in the morning. Accordingly, we observed that PAMP-triggered callose deposition was significantly higher in wild-type plants inoculated with *Pst* DC3000 *hrpA* in the subjective morning than in the evening, while no such temporal difference was evident in arrhythmic plants. Our results suggest that PAMP-triggered immune responses are modulated by the circadian clock and that temporal regulation allows plants to anticipate and respond more effectively to pathogen challenges in the daytime.

## Introduction

The circadian clock is an endogenous time-keeping mechanism that synchronizes biological processes with the external environment, such that they occur at optimal times of the day. In animals there is a growing body of evidence implicating the circadian clock in disease outcomes and the circadian regulation of immune responses. In Drosophila, circadian modulation of resistance to *Pseudomonas aeruginosa* and *Staphylococcus aureus* has been demonstrated, and clock mutants shown to display altered survival rates [1], [2]. While the role of light in the plant immune response is well established [3]–[5], the question as to whether the circadian clock plays a role in the outcome of plant-pathogen interactions has not been fully answered [6], [7]. The Arabidopsis central oscillator component CIRCADIAN CLOCK ASSOCIATED 1 (CCA1) was recently demonstrated to act as a positive integrator between the clock and defence pathways in resistance against an oomycete pathogen [8], but differences in host susceptibility to plant pathogens due to endogenously-driven circadian rhythms have not been demonstrated [8], [9].

Arabidopsis plants inoculated with avirulent *P. syringae* pv *maculicola* ES4326 *avrRpm1* (*Psm* ES4326 *avrRpm1*) 1 h after the beginning of the dark period displayed higher bacterial titres at 72 h post infection (hpi) than did plants inoculated at the beginning of the light period [9]. However, the authors attributed this to a direct effect of light rather than endogenous circadian effects on the induction of plant defense responses, as differences in salicylic acid (SA) production observed following infection with *Psm* ES4326 *avrRpm1* at two times of the day under a 9 h light/15 h dark cycle were not apparent under constant light or constant dark [9]. Similarly, inoculation of Arabidopsis with *Hyaloperonospora arabidopsidis* Emwa1 at dawn and dusk resulted in significantly higher levels of susceptibility, measured by sporangiophore counts, at dusk [8]. Nevertheless, bacterial titres and sporangiophore counts which represent the outcome of the plant-pathogen interaction were not determined under constant conditions in these experiments, and too few data points were used to rule out or confirm endogenous circadian clock regulation of plant defences.

The plant innate immune system is generally considered to consist of two branches [10], [11]. The first relies on the detection of evolutionary conserved pathogen associated molecular patterns (PAMPs) such as flagellin, chitin and lipopolysaccharide, by pattern recognition receptors at the plasma membrane [10]–[12]. Recognition events lead to the activation of PAMP-triggered immunity (PTI) which is associated with MAP-kinase signalling, induction of defence gene expression, production of reactive oxygen species, and callose deposition in the cell wall [13]. Induction of PTI is often sufficient to prevent microbial colonisation of the plant, however phytopathogens have evolved effectors which contribute to virulence in part by suppressing PTI, a phenomenon known as effector-triggered susceptibility (ETS) [10]. Plants, in turn have evolved a second branch of innate immunity which relies on the direct or indirect detection of these effector molecules. Recognition of an effector by the cognate host resistance (R) protein leads to activation of effector-triggered immunity (ETI), a quantitatively stronger response than PTI, often associated with the hypersensitive response (a form of programmed cell death) [12].

The circadian and diurnal regulation of much of the Arabidopsis transcriptome has been described [14], [15] and indeed defence-associated transcripts are among them [8], [16]. CCA1, a Myb-related transcription factor with a morning-phased expression of both transcript and protein, has been shown to regulate the expression of a number of defence genes [8], and binds to sequences in gene promoters called evening elements (EE) [17] to regulate their expression [18]. The rhythmic transcription of genes involved in defence may be due to co-localisation in the genome for efficient gene regulation as suggested for immunity genes in Drosophila [19], [20] or it may be for functional co-ordination, to prime defence responses at particular times of day when infections are most likely. Given the findings in Arabidopsis where only 63 of 3975 circadian regulated genes occurred in co-localised clusters in the genome [14], it seems that rhythmic transcription of genes involved in defence is more likely for functional co-ordination than being due to co-localisation.

In this study we investigated whether differences in Arabidopsis susceptibility to infection by *Pst* DC3000 are due to rhythms driven by the circadian clock, and what role the clock plays in the innate immune system to bring about temporal differences in the defence response. Our data suggest that the temporal variation in susceptibility of Arabidopsis to *Pseuodomonas syringae* results from clock-mediated modulation of PTI to ensure a maximal immune response to potential pathogens in the morning.

## Results

### Susceptibility of Arabidopsis to virulent *Pseudomonas syringae* varies with time of infection under constant light conditions

**Figure 1:**
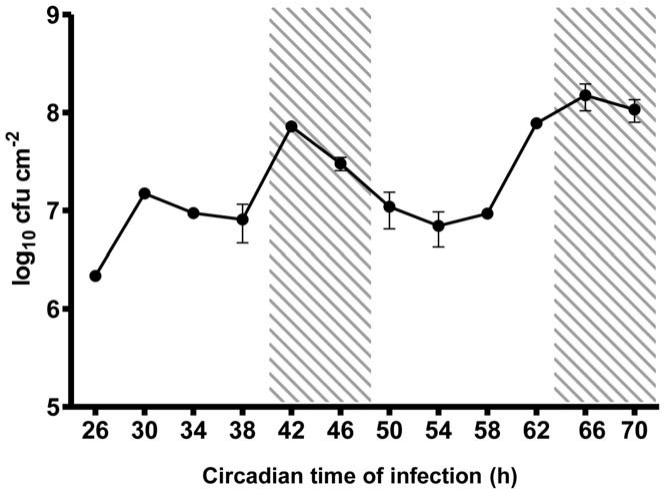
Arabidopsis displays temporal variation in susceptibility to *Pseudomonas syringae* infection. Bacterial growth in wild-type Columbia (Col-0) Arabidopsis was lowest when infected with *Pst* DC3000 in the subjective day, and highest when infected in the subjective night. *Pst* DC3000 counts (cfu cm^−2^ ± SEM, n = 3) in wild-type Arabidopsis leaves 48 hpi. Plants were grown in 16 h: light 8 h dark cycles at 22°C for 3 weeks prior to transfer to constant light at CT0. Infections were carried out in constant light at the times indicated. Shading indicates times of subjective night. General Linear Model (GLM) analysis revealed a significant effect of time of infection (p<0.001) on bacterial titres 48 hpi. Data shown are from one experiment representative of five independent experiments.

Previous observations that the susceptibility of a host to plant pathogens can vary depending on the time of day of infection have largely been attributed to the length of the light period following infection [9]. In order to exclude the effects of this variable on host susceptibility, we infected Arabidopsis Col-0 wild-type plants with the virulent pathogen *Pst* DC3000 (which triggers PTI but not ETI in Arabidopsis) at twelve time points over 48 h under free-running conditions of constant light. A consistent variation in host susceptibility with time of infection was observed in five independent experiments across two consecutive circadian cycles. Col-0 plants displayed greatest susceptibility to *Pst* DC3000 (measured as bacterial titre 48 hpi) when infected at circadian times (CT) 42 and 66 (subjective midnight) and greatest resistance at CT26 and 50 (subjective morning) (Figure 1). While these data are in broad agreement with those of Griebel & Zeier [9], the differential susceptibility of the host at different times of day cannot be attributed to light-dependent activation of defence responses, as all infections were carried out under constant conditions, and thus assayed after the same duration of light. However, these data are consistent with a role for the circadian clock in influencing the outcome of plant-pathogen interactions.

### Clock mutants do not exhibit temporal variation in susceptibility to virulent *Pseudomonas syringae*

**Figure 2:**
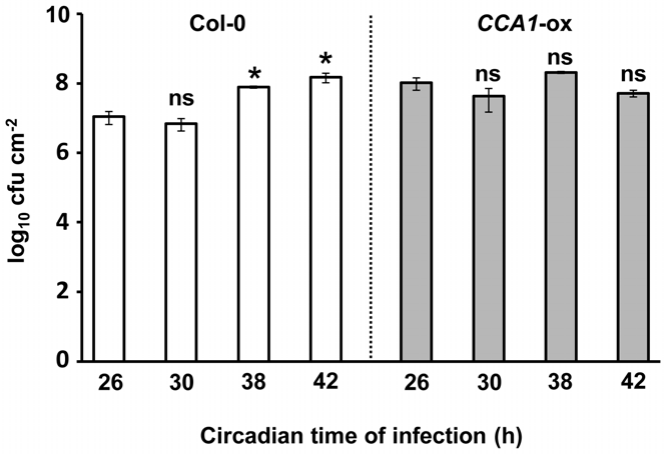
Temporal variation in susceptibility to *Pst DC3000* is abolished in plants over-expressing *CIRCADIAN CLOCK ASSOCIATED1*. *Pst* DC3000 counts (cfu cm^−2^ ± SEM, n = 3) in wild-type (open bars) versus *CCA1-*ox (shaded bars) Arabidopsis leaves 48 hpi. Plants were grown in 16 h: light 8 h dark cycles at 22°C for 3 weeks prior to transfer to constant light at CT0 and infected in free-running conditions at the times indicated. GLM analysis revealed significant effects of host genotype (p = 0.005) and time of infection (p<0.001) on bacterial titres at 48 hpi. A significant interaction between these two variables (p = 0.01) indicates that they combine non-additively to influence bacterial growth. * indicates a mean bacterial titre significantly different (p<0.05) from that observed at CT26 within each genotype (as determined by Bonferonni post-hoc analysis), while ns indicates no significant difference in titre to CT26. Data shown are from one experiment representative of four independent experiments.

**Figure 3:**
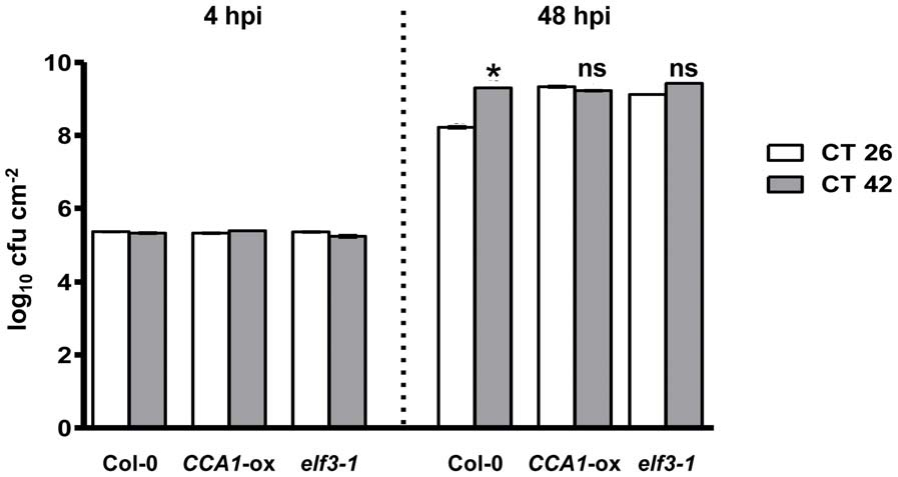
Disruption of circadian clock function causes loss of temporal variance in susceptibility to *Pst* DC3000. Plants were grown in 16 h: light 8 h dark cycles at 22°C for 3 weeks prior to transfer to constant light at CT0. Plants were infected at CT26 and CT42, the times of greatest variance in susceptibility in wild-type plants and bacterial titre determined at 48 hpi. Results are expressed as mean cfu cm^−2^ ± SEM (n = 3). GLM analysis revealed significant effects of host genotype (p<0.0001) and time of infection (p<0.0001) on bacterial titres at 48 hpi. A significant interaction between these two variables (p<0.0001) indicates that they combine non-additively to influence bacterial growth. * indicates a mean bacterial titre significantly different (p<0.05) from that observed at CT26 within each genotype (as determined by Bonferonni post-hoc analysis), while ns indicates no significant difference in titre to CT26. Data shown are from one experiment representative of three independent experiments.

If the clock plays a role in the host immune response, it would be expected that arrhythmic clock mutants should show altered susceptibility to plant pathogens. We first tested the susceptibility of the well characterised *CCA1*-overexpressing line (*CCA1-*ox) which is arrhythmic with respect to circadian outputs [21]. Col-0 and *CCA1*-ox plants were inoculated with *Pst* DC3000 at two time points in the subjective morning (CT26 and 30) and two time points in the subjective evening/night (CT38 and 42). While Col-0 plants again showed significantly higher bacterial titres when infected at CT38 and 42 compared to CT26, no such difference was observed in *CCA1*-ox plants (Figure 2). To exclude the possibility that the loss of temporal variation in susceptibility in *CCA1*-ox plants resulted simply from over-expression of the CCA1 transcription factor rather the absence of a functional circadian clock, we also tested the susceptibility of the *elf3-1* mutant (in the Col-0 background [22]), which displays arrhythmic circadian outputs under conditions of constant light [22], [23]. Plants were infected at the times that wild-type plants displayed the greatest resistance and susceptibility respectively, *viz.* CT26 and CT42. Bacterial titres were again significantly lower in Col-0 plants when infected at CT26 compared to CT42, while no differential response to time of infection was observed in either *elf3-1* or *CCA1*-ox (Figure 3). Indeed, bacterial titres in *elf3-1* and *CCA1*-ox plants at CT26 were not significantly different from those observed in Col-0 at CT42. These data indicate that the increased susceptibility of the clock mutants at CT26 was due to loss of temporal organisation (Figure 3), and suggested that a functional circadian clock was required for full activation of PTI against *Pst* DC3000 in the morning.

### Expression profiling reveals circadian oscillation of known defence genes in Arabidopsis

As a first step towards determining why a functional clock is required for a full PTI response to *Pst* DC3000 in the morning, we analysed publicly available microarray datasets to determine whether known components of the plant innate immune system display circadian oscillation in their expression patterns in Arabidopsis. Of the 121 defence-related genes (Table S1) analysed using Multi Experiment Viewer [24] and HAYSTACK [15], respectively 93 (77%) and 53 (44%) of these genes exhibited a diurnal expression pattern, with different genes showing peaks of expression at all phases of the cycle, although fewer genes peaked in the early evening than in other phases (Figure S1, Table S2). Most of the genes identified by HAYSTACK as having a diurnal expression pattern (48 out of 53) were also identified in the Multi Experiment Viewer analysis. Furthermore, 68 and 52 of the 121 defence-related genes displayed circadian expression under constant light conditions when clustered using Multi Experiment Viewer or analysed using HAYSTACK respectively and showed considerable overlap between analyses and with those that were diurnally regulated (Figure S2, Table S3).This demonstrates that defence responses are influenced by the circadian clock in the absence of pathogen challenge. Defence genes displaying both diurnal and circadian-regulated expression include the pattern recognition receptors for flagellin (*FLS2*) and EF-Tu (*EFR*), components of SA-mediated signalling (*ICS1*, *EDS1*, *EDS5* and *CPR5*) and jasmonic acid-mediated signalling (*COI1*), MAPK signalling components, WRKY transcription factors, and pathogenesis-related proteins.

**Figure 4:**
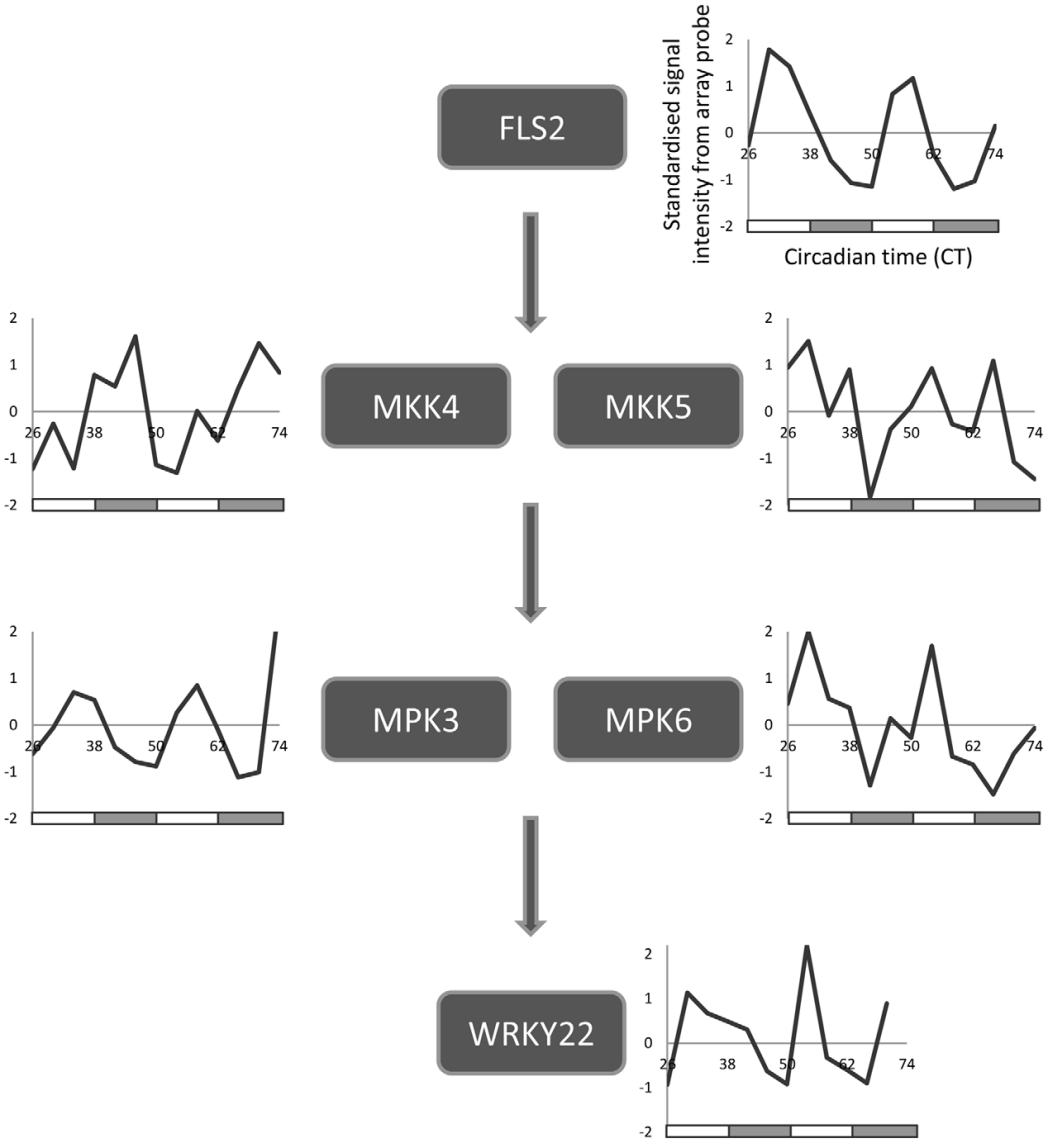
Circadian regulation of genes encoding the flagellin receptor (FLS2) and downstream MKK4/5-MAPK3/6-WRKY22 signalling module. Expression profiles were generated from standardised signal intensities from publicly available microarray datasets. With the exception of *MKK4* all genes exhibit peak expression in the subjective morning. No data are available for *WRKY29* expression levels in these datasets. Subjective day and night are indicated by white and grey boxes, respectively, below each plot.

Notably, we observed that a number of genes involved in the perception of the PAMP flagellin exhibit circadian regulation. Two MAPK signalling modules lie downstream of the flagellin pattern recognition receptor FLS2 [25]. All components of the MKK4/5-MAPK3/6-WRKY22 module [26] which is responsible for the activation of defence responses following flagellin detection display both diurnal and circadian regulation (Figure 4), with expression peaking during the day (with the exception of MKK4). In contrast, with the exception of MKK1, genes of the MEKK1-MKK1/2-MPK4 module [27] which acts to repress defence responses in the absence of pathogen attack, do not display circadian regulation. The morning peak in expression of the MKK4/5-MAPK3/6-WRKY22 module coincides with stomatal opening, a major point of entry for bacterial pathogens. Detection of PAMPs by the cognate pattern recognition receptors at the cell surface leads to activation of PTI, resulting in stomatal closure [28]. We therefore hypothesised that the Arabidopsis innate immune system may be primed by the circadian clock to respond most strongly to the detection of PAMPs in the morning.

### PAMP-triggered cell wall defence responses are modulated by the circadian clock

**Figure 5:**
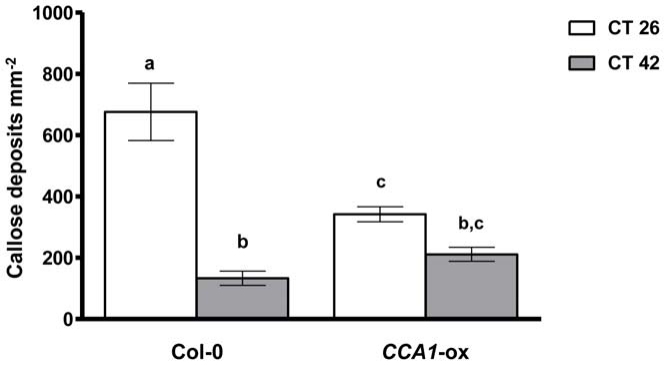
Disruption of clock function causes loss of temporal variance in callose deposition against *Pst* DC3000 *hrpA*. Callose deposits were counted from randomly selected regions of interest from Col-0 and *CCA1*-ox leaves infected with the *Pst* DC3000 *hrpA* mutant in constant light at CT26 and CT42. Results are expressed as average counts per mm^2^ ± SEM (n = 6). GLM analysis revealed significant effect of time of infection (p<0.0001), and a significant interaction between time of infection and host genotype (p<0.0001) on callose deposition. Mean callose deposits with different letters are significantly different (p<0.05) as determined by Bonferonni post-hoc analysis. Leaves mock treated with MgCl_2_ showed no callose deposition at either time point. Data shown are from one experiment representative of two independent experiments.

To investigate whether the infection time-dependent differences we observed in host susceptibility to *Pst* DC3000 might be attributable to modulation of PTI by the clock, we used callose deposition as a measure of the strength of the host PTI response following infection at CT26 or CT42, under constant light conditions. Callose is a complex high molecular weight glucan that is deposited during the cell wall modification process that occurs in response to PAMP detection in plants [13], [29]. Wild-type *Pst* DC3000 delivers effectors into the host cell during the infection process, where some function to subvert PTI responses, including cell wall modification [10], [30]. Thus in order to measure the strength of the PTI response at different times of infection or in different genetic backgrounds, rather than any differential effects of effector-triggered suppression of callose deposition, we infected plants with the *Pst* DC3000 *hrpA* mutant. This strain lacks a functional type III secretion system (rendering it non-pathogenic as it is unable to deliver effectors into the host), but is able to trigger a normal PTI response in Arabidopsis including callose deposition [31], [32]. We found that callose levels in Col-0 plants were significantly higher in plants infected with *Pst* DC3000 *hrpA* at CT26 than at CT42, while no such difference was evident in *CCA1*-ox (Figure 5). These data mirror the patterns observed in bacterial titres in Col-0 and *CCA1*-ox at CT26 and CT42 (Figures 2 & 3) and suggest that the ability of the host to mount this cell-wall based PTI response is modulated by the circadian clock.

**Figure 6:**
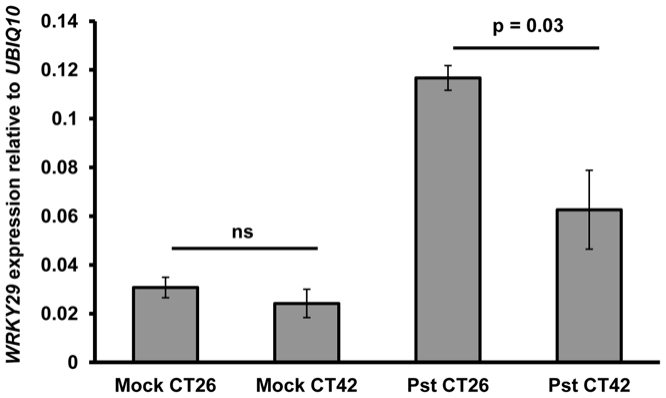
Induction of *WRKY29* expression in response to *Pst* DC3000 varies with time of infection. Col-0 plants were infected with *Pst* DC3000 (Pst) or 10 mM MgCl_2_ (mock) at CT26 or CT42 in constant light. Mean relative *WRKY29* expression levels (± SEM; n = 3) at 4 hpi (normalised to expression of the *UBIQ10* housekeeping gene) are shown. Student's t-test analysis indicated a significant difference in relative *WRKY29* expression between Col-0 plants infected with *Pst* DC3000 at CT26 and CT42.

In light of these results we attempted to determine whether earlier signalling events downstream of PAMP perception might also be subject to modulation by the circadian clock. Treatment of Arabidopsis protoplasts with flg22 leads to rapid induction of *WRKY29* expression [26]. We thus examined *WRKY29* expression in Col-0 plants 4 h after infection with *Pst* DC3000 at CT26 or CT42 using quantitative PCR. Relative *WRKY29* expression following *Pst* DC3000 infection was significantly higher in plants infected at CT26 than in plants infected at CT42 (Figure 6).

## Discussion

We have demonstrated, by infection of wild-type and arrhythmic plants over two circadian cycles in constant light, that differences in susceptibility to *Pst* DC300 are due to circadian clock regulation of the host immune response. Col-0 plants display greatest resistance to *Pst* DC3000 in the morning, and greatest susceptibility in the evening (Figure 1) under constant light conditions, while arrhythmic *CCA1*-ox and *elf3-1* plants do not show temporal variation in susceptibility to *Pst* DC3000 (Figures 2 & 3). Instead, bacterial titres observed in the morning in these arrhythmic plants are not significantly different to those observed in Col-0 plants at CT42, indicating that the increased resistance to *Pst* DC3000 observed in wild-type plants in the morning requires a functional circadian clock. Analysis of gene expression data revealed circadian regulation of the flagellin receptor FLS2 and the downstream MKK4/5-MAPK3/6-WRKY22 module, with expression peaking in the morning (Figure 4), suggesting that Arabidopsis may be primed to mount a stronger immune response following PAMP detection in the morning. In line with this hypothesis, we observed that levels of PAMP-triggered callose deposition were approximately 5-fold higher in Col-0 plants infected with *Pst* DC3000 *hrpA* in the subjective morning compared to those infected in the subjective evening under constant light conditions (Figure 5). In contrast, no significant difference in callose deposition was observed between subjective morning and evening in *CCA1*-ox plants (Figure 5), mirroring the patterns observed in bacterial titres in these plants (Figure 2 & 3). It is thus possible that the magnitude of the callose deposition response to PAMP detection in Col-0 plants is limited to a particular time of day through circadian gating. Circadian gating limits the time of maximal responsiveness to a particular signal, such as light, temperature or hormones [33]–[35], by altering the dynamics of signalling pathways or limiting the availability of specific factors. Circadian gating may also account for the higher *WRKY29* expression observed 4 hpi with *Pst* DC3000 in Col-0 plants infected at CT26 in comparison to CT42 (Figure 6). Together, these data suggest that the increased resistance to *Pst* DC3000 observed in the morning in Col-0 plants results from clock-mediated modulation of PTI.

Circadian clock regulation of PTI allows a stronger response during the day, correlating with the time of the circadian cycle when pathogens are most abundant [36] and the plant may be more prone to infection, due to open stomata which allow bacterial entry [28], [37]. While the recent study by Wang et al. [8] indicated that CCA1 enhanced resistance to an oomycete pathogen through activation of defence genes, we find that constitutive expression of CCA1 resulted in decreased resistance to *Pst* DC3000 during the day. Our findings indicate that the absence of temporal organisation of metabolism and development, and co-ordination of defence responses in this arrhythmic line may be of greater consequence than unregulated CCA1-mediated activation of defence genes. It has been suggested that the role of CCA1 in disease resistance demonstrated by Wang et al. [8] might thus be to act on an output pathway regulated by the clock, rather than through the central clock mechanism [7].

The finding that the promoters of many defence genes have EE [8] does not imply regulation solely by CCA1: the closely related Myb-like transcription factors LATE ELONGATED HYPOCOTYL (LHY), REVEILLE1 (RVE1) and RVE8 also bind to EE to regulate gene expression. While *cca1* mutants displayed increased susceptibility to *H. arabidopsidis* Emwa1, *lhy* mutants did not display a corresponding phenotype [8] which is an interesting and unexplained finding. LHY and CCA1 are partially redundant and act together at the same promoters [38]. RVE8, unlike CCA1 and LHY, has an evening phase of protein accumulation and thus regulates gene expression towards the end of the day [39]. RVE1 promotes auxin accumulation [40] which results in increased susceptibility to *Pst* DC3000 in Arabidopsis [41], [42] and auxin signalling is downregulated following PAMP detection [15], [42]. This complex regulation coupled with the increased susceptibility of both the arrhythmic *CCA1*-ox line and *elf3-1* mutant to *Pst* DC3000 infection demonstrated here, suggests that the central function of the circadian clock to integrate and co-ordinate metabolism and development is indeed required for plants to mount a full defence response to plant pathogens. Thus the circadian clock gives the host an adaptive advantage in the plant-pathogen interaction. The molecular mechanism by which the circadian modulation of PTI responses occurs is unknown. We suggest that genome-wide transcriptome profiling of Col-0 and clock mutants after pathogen infection at different times of day under constant light conditions would therefore be a worthwhile avenue for future research.

## Materials and Methods

### Plant Material

All plant lines used in pathogen growth assays were in the Columbia background (Col-0). Wild type *Arabidopsis thaliana* were from the Arabidopsis Biological Resource Centre and Seed Stock Centre (http://www.biosci.ohio-state.edu/pcmb/Facilities/abrc/abrchome.htm) while the *CCA1*-ox seeds were a gift from Dr Alex Webb (Department of Plant Sciences, University of Cambridge, UK) and *elf3-1* in the Col-0 background a gift from Dr Frank Harmon (University of California, Berkeley, USA).

### Plant growth conditions

*A. thaliana* seeds were surface sterilised and plated on Murashige and Skoog medium (Highveld Biological PTY, LTD, Lyndhurst, South Africa), solidified with 0.8% (w/v) agar. Seeds were stratified for 2 to 3 days at 4°C in the dark and thereafter transferred to a controlled environment with a long-day photoperiod (16 h light, 8 h dark) at 22°C and 55% relative humidity and cool white fluorescent light of 80–100 µmol m^−2^ s^−1^. Seven-day old seedlings were transferred to Imidocloprid-treated (‘Gaucho’, Bayer, Paarl, South Africa) peat (Jiffy Products, International AS, Norway) and vermiculite in a 1∶1 (v/v) ratio. Plants were transferred during the light period from long days to constant light (LL) (80–100 µmol m^−2^ s^−1^ from cool-white fluorescents) at least 24 h prior to pathogen infection.

### Pseudomonas syringae pv. tomato DC3000 infections of A. thaliana plants

Cultures of virulent *Pseudomonas syringae* pv. *tomato* DC3000 were grown to middle to late log phase in King's broth [43] supplemented with rifampicin (50 µg mL^−1^) at 30°C for 12 h before inoculation. All infections used bacteria that had been grown for the same length of time in liquid culture to avoid possible confounding factors due to differences in bacterial virulence with growth phase. The youngest fully expanded leaves of wild-type, *CCA1*-ox, and *elf3-1* plants were pressure infiltrated with either 1×10^6^ cfu mL^−1^ *Pst* DC3000 (Figure S3) or mock infected with 10 mM MgCl_2_ using a needle-less syringe into three marked leaves per plant [44]. In each experiment three plants were used per time point for each plant line. Pathogen infections were allowed to develop under constant light for the duration of the experiment.

### Bacterial growth assays

Bacterial growth assays were performed at 4 and 48 h post infection (hpi) to determine disease progression in the plants. Leaves were surface sterilised with 70% (v/v) ethanol and then washed in sterile water for 1 min before carrying out bacterial counts at each time point. A disc was cut out from each leaf using the base of a P200 Gilson pipette tip for each of the three leaves. The leaf discs were placed in 1 mL solution of 10 mM MgCl_2_ and crushed to release the bacteria. The resulting solution was serial diluted and 10 µL of each dilution was spot plated on KB plates containing 50 µg mL^−1^ rifampicin. The plates were incubated at 30°C for 48 h prior to counting of the colonies.

### Callose deposition assay

Four-week old *A. thaliana* Col-0 and *CCA1*-ox were entrained to long-days before transfer to constant light and were pressure infiltrated with 1×10^8^ cfu mL^−1^ *Pst* DC3000 *hrpA* bacteria at CT26 and CT42. Two leaves of each plant were infected; this was done in triplicate for each plant line per time point. Images were captured with a fluorescent inverted microscope (Nikon TMD-EF DIAPHOT-TMD, Tokyo, Japan). Aniline blue staining of callose was started 14 hpi and measured as described in Kim and Mackey [29].

### Quantitative PCR analysis of *WRKY29* expression

Four-week old *A. thaliana* Col-0 were entrained to long-days before transfer to constant light and were pressure infiltrated with 1×10^6^ cfu mL^−1^ *Pst* DC3000 or 10 mM MgCl_2_ (mock inoculation) at CT26 and CT42. Tissue was harvested at 4 hpi and total RNA extracted as previously described [45]. cDNA synthesis was carried out using ImpromII reverse transcription system (Promega), following the manufacturer's protocol except that half reaction-volumes and random hexamer primers were used. Real Time PCR was carried out using a Rotor Gene 6000 machine (Corbett Life Science, Sydney, Australia). The Dynamo flash SYBR Green qPCR kit (Finnzymes, Keilaranta, Finland) manufacturer's protocol was followed except half reaction volumes were used. A total reaction volume of 10 µL per reaction consisted of 1 µL of cDNA template, 0.5 µL each of forward and reverse primers (final concentration 0.5 µM each), 5 µL of SYBR green mix and 3 µL of sterile H_2_O. The PCR primers used were as follows, *UBIQ10*: 5′-TAAAAACTTTCTCTCAATTCTCTCT-3′ and 5′-TTGTCGATGGTGTCGGAGCTT-3′, and *WRKY29*: 5′-ATCCAACGGATCAAGAGCTG-3′ and 5′-GCGTCCGACAACAGATTCTC-3′. The relative standard curve method was used for quantification of *WRKY29* expression. R-squared values for the standard curves generated using purified PCR products were 0.997 and 0.996 for *UBIQ10* and *WRKY29* respectively. Relative *WRKY29* expression was determined by normalisation to the *UBIQ10* reference gene [21] in each biological replicate.

### Bioinformatic and statistical analyses

A list of 121 genes known to function in the host response to pathogen challenge in Arabidopsis was compiled (Table S1). The signal intensities for the corresponding ATH probes (Table S2) were extracted from normalised microarray data-sets that were obtained from the NCBI Gene expression omnibus (GEO) repository: GSE3416 (Diurnal regulation: examined by extracting RNA from plants entrained to 12 h/12 h light/dark cycle at 4 h intervals starting at the end of the night) [46] and GSE5612 (Circadian regulation: examined after plants entrained to 12 h/12 h light/dark cycle were exposed to continuous light. RNA was sampled at 4 h intervals over 2 d; made public in 2007 by Edwards K, Millar A, Townsend H, Emmerson Z, Schildknecht B). Signal intensities were standardised using the Gepas preprocessing tool [47] and analysed using MultiExperiment Viewer v4.6.1 [24]. Genes were clustered with the self-organising tree algorithm, using the Pearson Correlation distance metric, and classified according to patterns of expression and phase of peak expression time. Genes were considered to be rhythmically expressed if they displayed a single peak of expression under diurnal conditions, cycling under circadian conditions, or clustered together with a known clock- or clock controlled-gene. We also used the pattern matching algorithm HAYSTACK at http://haystack.cgrb.oregonstate.edu/ with models defined by Michaels et al. (2008) [15] to objectively determine which genes exhibited a circadian pattern of expression. General Linear Modelling analyses of all data were carried out using Statistica (version 9). Raw data were transformed prior to GLM analysis using natural logs for bacterial titres (Figures 1, 2 & 3), and square root values for callose deposits (Figure 5) to ensure homogeneity of variance and normality of error. Where the effect of two independent variables (time of infection and host genotype) on a single dependent variable (bacterial titre or callose deposition) was analysed, an interaction term was included in the GLM model to test whether the independent variables combined non-additively to effect the dependent variable.

## Supporting Information

Figure S1 **121 defence genes (plus** ***CCA1*** **and** ***LHY*** **) were clustered using MeV v4.6.1 according to their expression profile (GEO accession 3416) across one diurnal cycle (12 h light/12 h dark).** Genes in clusters with green numbers were considered to show diurnal regulation. See Table S2 for lists of genes in each of these clusters.
(PDF)

Figure S2 **121 defence genes (plus** ***CCA1*** **and** ***LHY*** **) were clustered using MeV v4.6.1 according to their expression profile (GEO accession 5612) under constant light conditions.** Genes in clusters with green numbers were considered to show circadian regulation. See Table S3 for lists of genes in each of these clusters.
(PDF)

Figure S3 **Bacterial titres at 4 hpi (± SEM, n = 3) following infection with 10^6^ cfu mL** ^−1^ ***Pst* DC3000 (corresponding to 10^4^ colony forming units cm^−2^).** Plants were grown in 16 h: light 8 h dark cycles at 22°C for 3 weeks prior to transfer to constant light at CT0. Infections were carried out in constant light at the times indicated.
(PDF)

Table S1 **List of 121 defence-related genes analysed for diurnal and circadian transcriptional regulation using publically available microarray data.** *CCA1* and *LHY* are included as known genes subject to circadian regulation.
(PDF)

Table S2 **Genes exhibiting diurnal expression pattern.** Genes highlighted in blue also display circadian patterns of expression. Known circadian genes *CCA1* and *LHY* are highlighted in red. Genes determined to have diurnal expression by both Multi Experiment Viewer clustering and HAYSTACK are indicated in bold. The cluster into which each gene falls (in Figure S1) and the phase of transcript accumulation according to HAYSTACK is indicated.
(PDF)

Table S3 **Genes exhibiting circadian expression pattern.** Genes highlighted in yellow also display diurnal patterns of expression. Known circadian genes *CCA1* and *LHY* are highlighted in red. Genes determined to have circadian expression by both Multi Experiment Viewer clustering and HAYSTACK are indicated in bold. The cluster into which each gene falls (in Figure S2) and the phase of transcript accumulation according to HAYSTACK is indicated.
(PDF)
