# Supplementary material for: Defence Responses of Arabidopsis thaliana to Infection by Pseudomonas syringae Are Regulated by the Circadian Clock
Source: PLoS One. 2011 Oct 31;6(10):e26968. doi: 10.1371/journal.pone.0026968 (PMC3205005; doi:10.1371/journal.pone.0026968)

Figure S1: 121 defence genes (plus *CCA1* and *LHY*) were clustered using MeV v4.6.1 according to their expression profile (GEO accession 3416) across one diurnal cycle (12 h light/12 h dark). Genes in clusters with green numbers were considered to show diurnal regulation. See Table S2 for lists of genes in each of these clusters.

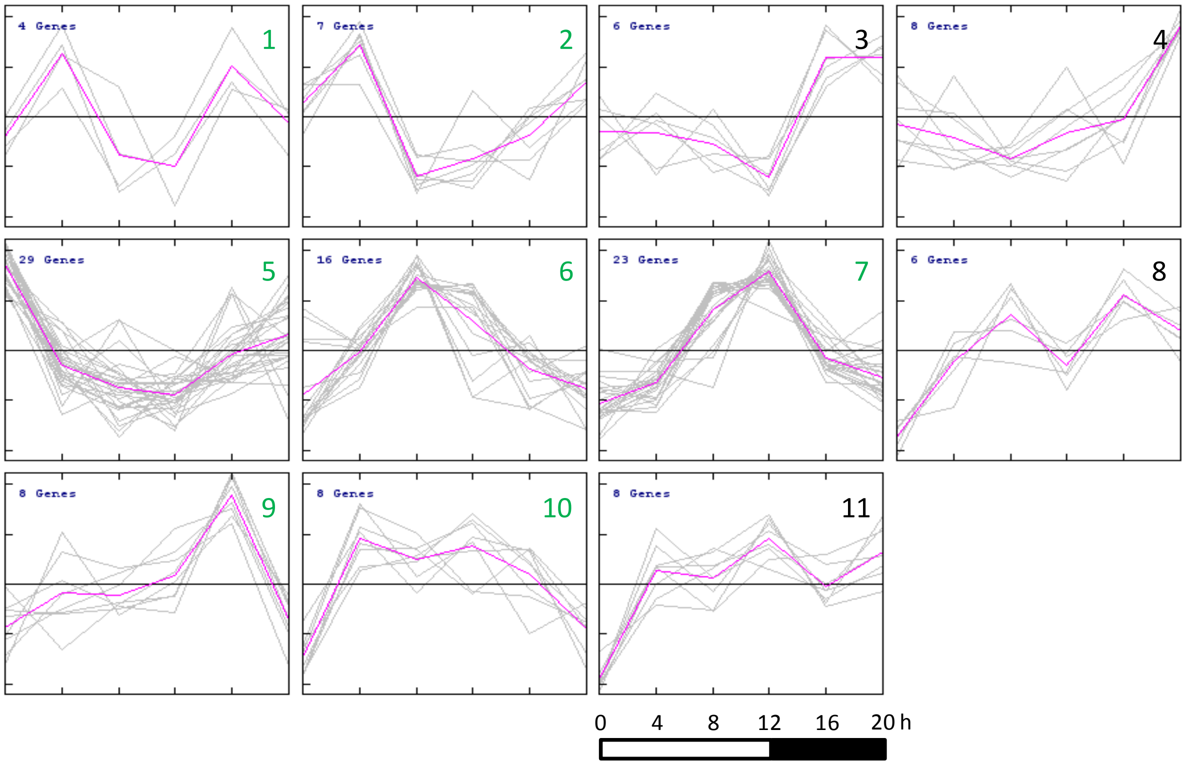

Supplement: Figure S1 — 121 defence genes (plus CCA1 and LHY ) were clustered using MeV v4.6.1 according to their expression profile (GEO accession 3416) across one diurnal cycle (12 h light/12 h dark). Genes in clusters with green numbers were considered to show diurnal regulation. See Table S2 for lists of genes in each of these clusters. (PDF) [file pone.0026968.s001.pdf]
