# Supplementary material for: Defence Responses of Arabidopsis thaliana to Infection by Pseudomonas syringae Are Regulated by the Circadian Clock
Source: PLoS One. 2011 Oct 31;6(10):e26968. doi: 10.1371/journal.pone.0026968 (PMC3205005; doi:10.1371/journal.pone.0026968)

Figure S2: 121 defence genes (plus *CCA1* and *LHY*) were clustered using MeV v4.6.1 according to their expression profile (GEO accession 5612) under constant light conditions. Genes in clusters with green numbers were considered to show circadian regulation. See Table S3 for lists of genes in each of these clusters.

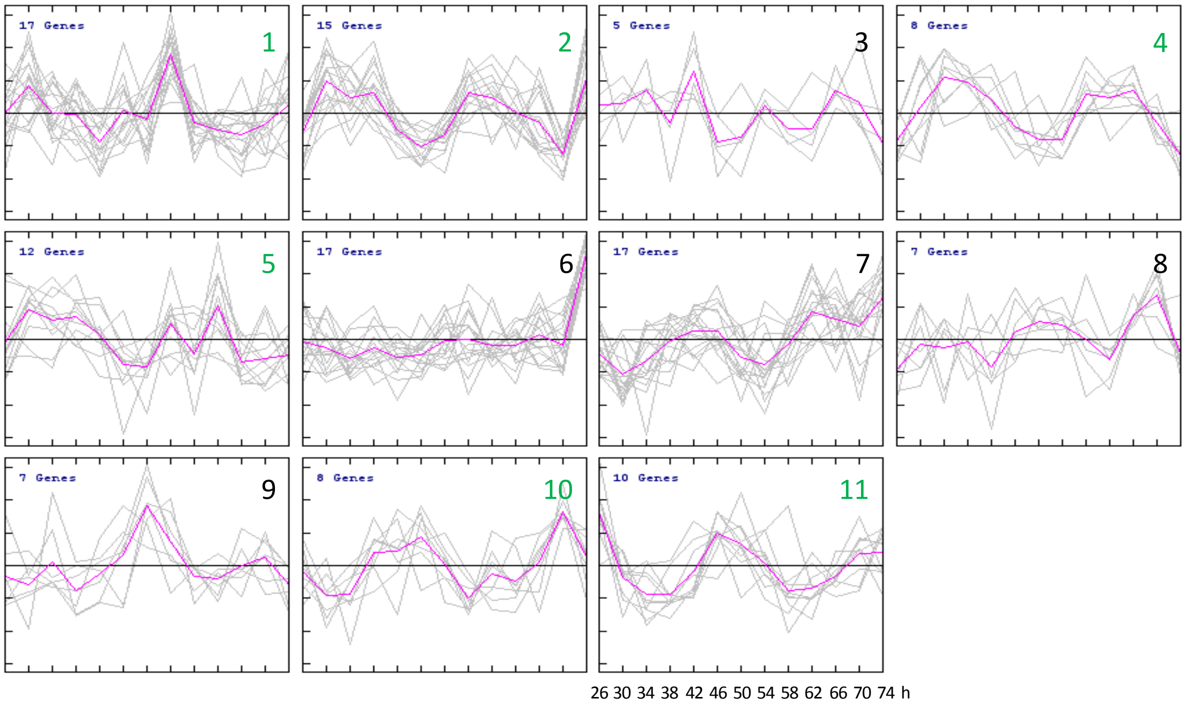

Supplement: Figure S2 — 121 defence genes (plus CCA1 and LHY ) were clustered using MeV v4.6.1 according to their expression profile (GEO accession 5612) under constant light conditions. Genes in clusters with green numbers were considered to show circadian regulation. See Table S3 for lists of genes in each of these clusters. (PDF) [file pone.0026968.s002.pdf]
