# Supplementary material for: Defence Responses of Arabidopsis thaliana to Infection by Pseudomonas syringae Are Regulated by the Circadian Clock
Source: PLoS One. 2011 Oct 31;6(10):e26968. doi: 10.1371/journal.pone.0026968 (PMC3205005; doi:10.1371/journal.pone.0026968)

Figure S3: Bacterial titres (± SEM, n =3) at 4 hpi following infection with 10^6 cfu mL^{-1} *Pst* DC3000 (corresponding to 10^4 colony forming units cm^{-2}). Plants were grown in 16 h: light 8 h dark cycles at 22 °C for 3 weeks prior to transfer to constant light at CT 0. Infections were carried out in constant light at the times indicated.

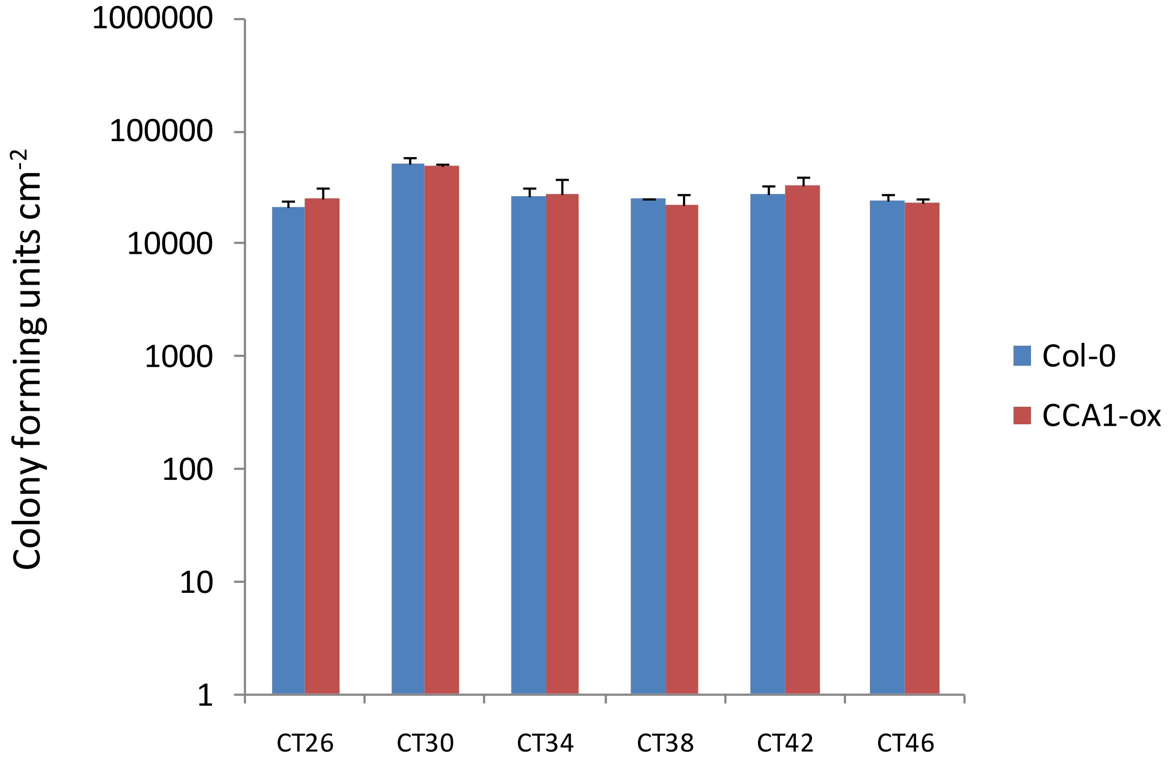

Supplement: Figure S3 — Bacterial titres at 4 hpi (± SEM, n = 3) following infection with 106 cfu mL −1 Pst DC3000 (corresponding to 104 colony forming units cm−2). Plants were grown in 16 h: light 8 h dark cycles at 22°C for 3 weeks prior to transfer to constant light at CT0. Infections were carried out in constant light at the times indicated. (PDF) [file pone.0026968.s003.pdf]
