# Supplementary material for: Defence Responses of Arabidopsis thaliana to Infection by Pseudomonas syringae Are Regulated by the Circadian Clock
Source: PLoS One. 2011 Oct 31;6(10):e26968. doi: 10.1371/journal.pone.0026968 (PMC3205005; doi:10.1371/journal.pone.0026968)
Supplement: Table S1 — List of 121 defence-related genes analysed for diurnal and circadian transcriptional regulation using publically available microarray data. CCA1 and LHY are included as known genes subject to circadian regulation. (PDF) [file pone.0026968.s004.pdf]

**Table S1:** List of 121 defence-related genes analysed for diurnal and circadian transcriptional regulation using publically available microarray data. *CCA1* and *LHY* are included as known genes subject to circadian regulation.

| LOCUS ID | GENE NAME | FUNCTION | REFS |
|---|---|---|---|
| AT2G46830 | CIRCADIAN CLOCK ASSOCIATED 1 (CCA1) | MYB transcription factor, CCA1 and LHY function synergistically in regulating circadian rhythms of Arabidopsis | [1] [2] |
| AT1G01060 | LATE ELONGATED HYPOCOTYL (LHY) | As above | [1] [2] |
| AT2G14610 | PATHOGENESIS-RELATED GENE 1 (PR1) | expression is induced in response to a variety of pathogens and SA. It is a useful molecular marker for the systemic acquired resistance (SAR) response. | [3] [4] [5] |
| AT3G57260 | PR2 | beta 1,3-glucanase, systemic acquired resistance | [5] |
| AT1G75040 | PR5 | Thaumatin-like protein involved in response to pathogens. | [5] |
| AT1G08450 | CALRETICULIN 3 (CRT3) | non-receptor component required for EFR-mediated immunity. defense response to bacterium | [6] |
| AT5G64930 | CONSTITUTIVE EXPRESSION OF PR GENES 5 (CPR5) | regulator of expression of PR genes. Participates in signal transduction pathways involved in plant defense (SAR). | [7] [8] [9] |
| AT4G39030 | ENHANCED DISEASE SUSCEPTIBILITY 5 (EDS5);SA INDUCTION DEFICIENT 1 (SID1) | orphan multidrug and toxin extrusion transporter. Essential component of salicylic acid-dependent signaling for disease resistance. Member of the MATE-transporter family. Expression induced by salicylic acid | [10] |
| AT1G74710 | EDS16 / ISOCHORISMATE SYNTHASE 1 (ICS1) | SA biosynthesis | [11] |
| AT3G48090 | EDS1 | encodes lipase-like protein that function in resistance (R) gene-mediated and basal plant disease resistance, required for accumulation of salicylic acid | [12] [13] |
| AT1G64280 | NON-EXPRESSER OF PR GENES 1 (NPR1) | Key regulator of SA-mediated signaling | [14] |
| AT3G52430 | PHYTOALEXIN DEFICIENT 4 (PAD4) | encodes lipase-like protein that function in resistance (R) gene-mediated and basal plant disease resistance, required for accumulation of salicylic acid | [12] [15] [16] |
| AT4G23100 | PAD2 / GLUTAMATE-CYSTEINE LIGASE (GSH1) | catalyzes the first, and rate-limiting, step of glutathione biosynthesis. | [17] [18] |
| AT3G53260 | PAL2 | phenylpropanoid biosynthesis, SA biosynthesis | [19] [20] |
| AT5G04230 | PAL3 | as above | [19] [20] |
| AT3G10340 | PAL4 | as above | [20] [21] |
| AT5G15410 | DEFENSE NO DEATH 1 (DND1) | conducts Ca2+ into cells which is linked to the generation of NO and the NO signaling pathway involved in the innate immune | [22] |

| | | | |
|---|---|---|---|
| | | response to pathogens defense response | |
| AT1G08720 | ENHANCED DISEASE RESISTANCE 1 (EDR1) | confers resistance to powdery mildew disease caused by the fungus Erysiphe cichoracearum, response to bacterium | [23 24] |
| AT2G31880 | SUPPRESSOR OF BIR1 1 (SOBIR1) | leucine rich repeat transmembrane protein that is expressed in response to Pseudomonas syringae. positive regulation of defense response | [25] |
| AT5G10380 | RING1 | RING finger domain protein with E3 ligase activity. Expression is increased in response to fungal pathogen. | [26] |
| AT5G46330 | FLAGELLIN-SENSITIVE 2 (FLS2) | essential in the perception of flagellin, involved in MAPK signalling relay involved in innate immunity. | [27] |
| AT5G20480 | EF-TU RECEPTOR (EFR) | LRR receptor kinase (LRR-RLK). Functions as the receptor for bacterial PAMP (pathogen associated molecular patterns) EF-Tu | [28] |
| AT3G21630 | Chitin Elicitor Receptor Kinase 1 (CERK1) | LysM receptor-like kinase. Essential in the perception and transduction of the chitin oligosaccharide elicitor. Involved in chitin-mediated plant innate immunity. Located in the plasma membrane. | [29] |
| AT3G11820 | PENETRATION (PEN)-1 / SYNTAXIN OF PLANTS 121 (SYP121) | plasma membrane localized syntaxin, member of the SNARE superfamily proteins, Required for MLO resistance, SA mediated signaling pathway | [30 31 32 33] |
| AT2G44490 | PEN2 | peroxisomal glucosyl hydrolase, component of an inducible preinvasion resistance mechanism. Required for MLO resistance. | [34] |
| AT1G59870 | PEN3 | plasma membrane ABC transporter, contributes to nonhost resistance to inappropriate pathogens that enter by direct penetration in a salicylic acid–dependent manner. Required for MLO resistance. | [35] |
| AT1G01720 | ATAF1 | putative transcriptional activator with NAC domain. Transcript level increases in response to wounding and abscisic acid. | [36] |
| AT1G61560 | MILDEW RESISTANCE LOCUS O 6 (MLO6) | homolog of the barley mildew resistance locus o (MLO) protein, induced in response to a broad range of pathogens, confirmed co-expression with disease resistant protein encoding genes | [37 38] |
| AT2G39200 | MLO12 | homolog of the barley mildew resistance locus o (MLO) protein, defense response to fungus, incompatible interaction | [39] |
| AT1G11310 | MLO2 | homolog of the barley mildew resistance locus o (MLO) protein, defense response to fungus, incompatible interaction | [39] |
| AT3G54920 | POWDERY MILDEW RESISTANT 6 (PMR6) | Powdery mildew resistant mutant encodes a pectate lyase-like protein, defense response, incompatible interaction | [40] |
| AT5G58600 | PMR5 | Involved in resistance to the powdery mildew species Erysiphe cichoracearum and Erysiphe orontii, but not to the unrelated pathogens Pseudomonas syringae or Peronospora parasitica. | [41] |
| AT5G24530 | DOWNY MILDEW | putative 2OG-Fe(II) oxygenase that is defense-associated but | [42] |

| | | | |
|---|---|---|---|
| | RESISTANT 6 (DMR6) | required for susceptibility to downy mildew,response to bacterium | |
| AT1G04750 | VESICLE-ASSOCIATED MEMBRANE PROTEIN 721 (VAMP721) | forms part of plasma membrane SNARE PEN1-SNAP33-VAMP721/722 secretory complex that functions in plant defence | [43 44 45] |
| AT5G61210 | SOLUBLE N-ETHYLMALEIMIDE-SENSITIVE FACTOR ADAPTOR PROTEIN 33 (SNAP33) | Forms part of plasma membrane SNARE PEN1-SNAP33-VAMP721/722 secretory complex that functions in plant defence | [43 46] |
| AT5G07880 | SYNAPTOSOMAL-ASSOCIATED PROTEIN SNAP25-LIKE 29 (SNAP29) | SNAP receptor activity | [30] |
| AT1G13890 | SNAP30 | SNAP receptor activity | [30] |
| AT4G03550 | GLUCAN SYNTHASE-LIKE 5 (ATGSL05), POWDERY MILDEW RESISTANT 4 (PMR4) | Encodes a callose synthase that is required for wound and papillary callose formation in response to fungal pathogens.. Contributes to PAMP-induced basal defense, callose deposition in cell wall during defense response | [17] |
| AT1G33960 | AVRRPT2-INDUCED GENE 1 (AIG1) | induced by avirulence gene avrRpt2 and RPS2 after infection with Pseudomonas syringae carrying avrRpt2 | [47] |
| AT3G28930 | AVRRPT2-INDUCED GENE 2 (AIG2) | AVRRPT2-INDUCED GENE 2 (AIG2) | [47] |
| AT4G26090 | RESISTANT TO P. SYRINGAE 2 (RPS2) | disease resistance protein (CC-NBS-LRR class). Encodes a plasma membrane protein that confers resistance to P syringae infection by interacting with the avirulence gene avrRpt2. RPS2 protein interacts directly with plasma membrane associated protein RIN4 and this interaction is disrupted by avrRpt2. | [48] |
| AT3G07040 | RESISTANCE TO P. SYRINGAE PV MACULICOLA 1 (RPM1) | disease resistance protein(CC-NBS-LRR class), Confers resistance to P syringae strains that carry the avirulence genes avrB and avrRpm1. | [49] |
| AT3G25070 | RPM1 INTERACTING PROTEIN 4 (RIN4) | Encodes a member of the R protein complex and may represent a virulence target of type III pili effector proteins (virulence factors) from bacterial pathogens, which is 'guarded' by R protein complex (RPM1 and RPS2 proteins). | [50] |
| AT5G45250 | RESISTANT TO P. SYRINGAE 4 (RPS4) | Toll/interleukin-1 receptor (TIR)-nucleotide binding site (NBS)-LRR class of disease resistance (R ) genes. Confers specific resistance to Pseudomonas syringae pv. tomato carrying the avirulence gene AvrRPS4. | [51] |
| AT1G12220 | RESISTANT TO P. SYRINGAE 5 (RPS5) | Resistance gene, Confers resistance to Pseudomonas syringae strains that express avrPphB. | [52] |
| AT5G13160 | avrPphB susceptible 1 (PBS1) | Mutant is defective in perception of Pseudomonas syringae avirulence gene avrPphB. Encodes a putative serine-threonine kinase. | [52] |

| | | | |
|---|---|---|---|
| AT5G14930 | SENESCENCE-ASSOCIATED GENE 101 (SAG101), | EDS1-interacting acyl-hydrolase | 53 54 |
| AT1G33560 | ACTIVATED DISEASE RESISTANCE 1 (ADR1) | Encodes a NBS-LRR disease resistance protein that possesses N-terminal kinase subdomains. Activation tagged mutant of ADR1 showed elevated levels of SA and reactive oxygen species in addition to number of defense gene transcripts. Exhibits resistance to number of microbial pathogens. | 55 |
| AT4G33300 | ADR1-like 1 (ADR1-L1), putative disease resistance protein | disease resistance protein (CC-NBS-LRR class) | 56 |
| AT3G50480 | HOMOLOG OF RPW8 4 (HR4) | RPW8 (RESISTANCE TO POWDERY MILDEW 8) is a disease resistance (R) gene involved in the recognition of a broad range of powdery mildew pathogens and induces localized SA-dependent defenses. | 57 58 |
| AT2G26560 | PHOSPHOLIPASE A 2A (PLA2A) | Encodes a lipid acyl hydrolase with wide substrate specificity that accumulates upon infection by fungal and bacterial pathogens. | 59 |
| AT3G04720 | PATHOGENESIS-RELATED 4 (PR4) | Encodes a protein similar to the antifungal chitin-binding protein hevein from rubber tree latex. mRNA levels increase in response to ETH and turnip crinkle virus infection. | 60 |
| AT3G12500 | BASIC CHITINASE (CHIB) | encodes a basic chitinase involved in ETH/JAmediated signalling pathway during systemic acquired resistance defense response to fungus | 61 |
| AT4G11650 | osmotin 34 (ATOSM34) | defense response to bacterium and fungas, incompatible interaction | 62 |
| AT1G73680 | ALPHA DIOXYGENASE (ALPHA DOX2) | initiate the synthesis of oxylipin, pathogen-responsive alpha-dioxygenase, putative, response to other organism | 63 |
| AT1G21250 | WAK1 | may function as a signaling receptor of extracellular matrix component such as oligogalacturonides. expression induced by pathogens, or SA in an NPR1 dependent manner, required for resistance to lethal SA levels. | 64 |
| AT1G79680 | WAKL10 | Induced in response to a broad range of pathogens, confirmed co-expression with disease resistant protein encoding genes | 38 |
| AT1G79670 | RESISTANCE TO FUSARIUM OXYSPORUM 1 (RFO1), WAKL22 | A novel type of dominant disease-resistance protein that confers resistance to a broad spectrum of Fusarium races | 65 |
| AT5G47910 | RESPIRATORY BURST OXIDASE HOMOLOGUE D (RBOHD) | Interacts with AtrbohF gene to fine tune the spatial control of ROI production and hypersensitive response to cell in and around infection site | 66 |
| AT1G64060 | RESPIRATORY BURST OXIDASE PROTEIN F | Interacts with AtrbohD gene to fine tune the spatial control of ROI production and hypersensitive response to cell in and | 67 |

| | | | |
|---|---|---|---|
| | (ATRBOH F) | around infection site | |
| AT2G39940 | CORONATINE INSENSITIVE 1 (COI1) | Required for wound- and jasmonates-induced transcriptional regulation, defense response to fungus | [68] |
| AT2G44050 | COI1 SUPPRESSOR1 (COS1) | JA mediated signaling pathway | [69] |
| AT5G11270 | OVEREXPRESSOR OF CATIONIC PEROXIDASE 3 (OCP3) | mediates resistance to infection by necrotrophic pathogens, JA mediated signaling pathway | [70] |
| AT2G46370 | JASMONATE RESISTANT 1 (JAR1) | jasmonate-amino synthetase activity, catalyzes the formation of a biologically active jasmonyl-isoleucine (JA-Ile) conjugate, response to JA stimulus | [71] |
| AT5G42650 | ALLENE OXIDE SYNTHASE (AOS) | JA biosynthetic pathway, defense response | [72 73] |
| AT2G06050 | OPDA-REDUCTASE 3 (OPR3) | JA biosynthetic process | [74] |
| AT4G16760 | ACYL-COA OXIDASE 1 (ACX1) | JA biosynthetic process | 75 |
| AT1G19640 | JA CARBOXYL METHYLTRANSFERASE (JMT) | JA biosynthetic process | 76 |
| AT1G19670 | CORONATINE-INDUCED PROTEIN 1 (ATCLH1), | Chlorophyllase 1, chlorophyll catabolic process, response to stress and JA stimulus | 77 |
| AT4G23600 | CORONATINE INDUCED 1 (CORI3) | generation of precursors of ETH biosynthesis, response to JA stimulus | [78 79] |
| AT1G75830 | PLANT DEFENSIN 1.2 (PDF1.1) / LOW-MOLECULAR-WEIGHT CYSTEINE-RICH 67 (LCR67) | Predicted to encode a PR (pathogenesis-related) protein, belongs to the plant defensin (PDF) family protein, defense response | [80] |
| AT2G26020 | PDF1.2b | As above | [80] |
| AT1G19610 | PDF1.4 / LCR78 | As above | [80] |
| AT2G02120 | PDF2.1 / LCR70 | As above | [80] |
| AT2G02100 | PDF2.2 / LCR69 | As above | [80] |
| AT2G02130 | PDF2.3 / LCR68 | As above | [80] |
| AT1G61070 | PDF2.4 / LCR66 | As above | [80] |
| AT5G63660 | PDF2.5 / LCR74 | As above | [80] |
| AT2G02140 | PDF2.6 / LCR72 | As above | [80] |
| AT5G38330 | LCR80 | As above | [80] |

| | | | |
|---|---|---|---|
| AT5G44420 | PDF1.2 / LCR77 | Encodes an ETH- and jasmonate-responsive plant defensin. mRNA levels are not responsive to SA treatment; although jasmonate and SAcan act synergistically to enhance the expression of this gene. Belongs to the plant defensin (PDF) family. | [81 82] |
| AT1G72260 | THIONIN 2.1 (THI2.1) | Encodes a thionin which is a cysteine rich protein having antimicrobial properties. Thi2.1 is expressed in response to a variety of pathogens and induced by ETH and JA Belongs to the plant thionin (PR-13) family | [83] |
| AT5G36910 | THIONIN 2.2 (THI2.2) | Predicted to encode a PR, belongs to the plant defensin (PDF) family protein, defense response defense response | [80] |
| AT2G33150 | PEROXISOMAL 3-KETOACYL-COA THIOLASE 3 (PKT3), KAT 2 | JA biosynthetic process | [84 85] |
| AT1G04710 | PKT4 | acetyl-CoA C-acyltransferase | [84 85] |
| AT4G23570 | SGT1A, | Closely related to SGT1B, may function in SCF(TIR1) mediated protein degradation. AtSGT1a and AtSGT1b are functionally redundant in the resistance to pathogens. | [86] |
| AT4G11260 | SGT1B | as above, required for defense signaling conferred by several downy mildew resistance genes, JA mediated signaling pathway | [87] |
| AT5G48485 | DEFECTIVE IN INDUCED RESISTANCE 1 (DIR1) | encodes a putative apoplastic lipid transfer protein that is involved in SAR | [88] |
| AT3G45640 | MITOGEN-ACTIVATED PROTEIN KINASE 3 (MPK3) | Functions in MAP kinase cascade involving MEKK1, MKK4/MKK5 and MPK3/MPK6 that functions downstream of the FLS2 fagellin receptor, activation of this MAPK cascade confers resistance to both bacterial and fungal pathogens | [89 90] |
| AT4G01370 | MPK4 | nuclear and cytoplasmically localized MAP kinase, regulates SA and jasmonic acid/ETH-dependent responses via EDS1 and PAD4 | [91] |
| AT2G43790 | MPK6 | as MPK3 | [89 92] |
| AT4G26070 | MITOGEN-ACTIVATED PROTEIN KINASE KINASE 1 (MKK1) / MEK1 (MAP KINASE/ ERK KINASE 1) | Shares functional redundancy with MKK1/2, involved in jasmonate- and salicylate-dependent defense responses | [93] |
| AT4G29810 | MKK2 | Shares functional redundancy with MKK1/2, involved in jasmonate- and salicylate-dependent defense responses | [93] |
| AT1G51660 | MKK4 | as MPK3 | [89] |
| AT3G21220 | MKK5 | as MPK3 | [89] |
| AT1G08720 | ENHANCED DISEASE RESISTANCE 1 (EDR1); MAP kinase kinase kinase/ kinase/ protein serine/threonine/tyrosine | confers resistance to powdery mildew disease caused by the fungus Erysiphe cichoracearum, response to bacterium and fungus | [23 24] |

| | | | |
|---|---|---|---|
| | kinase | | |
| AT1G73500 | MAP KINASE KINASE 9 (MKK9) | Autophosphorylates and also phosphorylates MPK3 and MPK6. Independently involved in ETH and calmalexin biosynthesis. Induces transcription of ACS2, ACS6, ERF1, ERF2, ERF5, ERF6, CYP79B2, CYP79B3, CYP71A13 and PAD3. | [94] |
| AT3G06110 | MAPK PHOSPHATASE 2 (MKP2) | nuclear-localized MAPK phosphatase. Plants with reduced levels of MKP2 transcripts are hypersensitive to ozone and ozone-mediated activation of MPK3 and MPK6 is prolonged in these plants. | [95] |
| AT5G53210 | SPEECHLESS (SPCH) | SPCH is a substrate of a kinase MPK3 and MPK6 | [96] |
| AT1G10210 | MITOGEN-ACTIVATED PROTEIN KINASE 1 (ATMPK1) | Activated by wounding JA | [97] |
| AT1G18350 | MAP KINASE KINASE7 (ATMKK7) | defense response to bacterium, positively regulates plant basal and systemic acquired resistance (SAR) | [98] |
| AT4G08500 | MAP KINASE KINASE KINASE 1 (MEKK1) | as MPK3 | [89 99] |
| AT1G19180 | JASMONATE-ZIM-DOMAIN PROTEIN 1 (JAZ1); (TIFY10A) | nuclear-localized protein involved in jasmonate signaling, induced by jasmonate | [100 101 102] |
| AT1G74950 | JAZ2/TIFY10B | response to JA stimulus and wounding | [102 103] |
| AT3G17860 | JAZ3 | negatively regulates the key transcriptional activator of JA responses, AtMYC2, induced by jasmonate | [100 102] |
| AT5G13220 | JAZ10 | induced by jasmonate, regulation of systemic acquired resistance | [104 105] |
| AT1G32640 | MYC2 | MYC-related transcriptional activator, regulates diverse JA-dependent functions. | [81] |
| AT4G17500 | ETH RESPONSIVE ELEMENT BINDING FACTOR 1 (ATERF-1) | member of the ERF (ETH response factor) subfamily B-3 of ERF/AP2 transcription factor family, response to chitin | [26] |
| AT3G23240 | ETH RESPONSE FACTOR 1 (ERF1) | member of the ERF subfamily B-3 of ERF/AP2 transcription factor Involved in ETH signaling cascade,downstream of EIN2 and EIN3. | [106] |
| AT3G16770 | ETH-RESPONSIVE ELEMENT BINDING PROTEIN (ATEBP) | member of the ERF subfamily B-3, part of the ETH signaling pathway and is predicted to act downstream of EIN2 and CTR1, but not under EIN3. response to other organism, ETH mediated signaling pathway | [107] |
| AT3G15210 | ETH RESPONSIVE ELEMENT BINDING FACTOR 4 (ERF4) | member of the ERF subfamily of transcription factors, negative regulator of JA-responsive defense gene expression and resistance to the necrotrophic fungal pathogen Fusarium oxysporum and antagonizes JA inhibition of root elongation | [108] |

| | | | |
|---|---|---|---|
| AT5G03280 | ETH INSENSITIVE 2 (EIN2) | Involved in ETH signal transduction. Acts downstream of CTR1, defense response to fungus | [109] |
| AT1G66340 | ETH RESPONSE 1 (ETR1) | ETH receptor, defense response to bacterium | [110] |
| AT2G19560 | ENHANCED ETH RESPONSE 5 (EER5) | encodes a protein with a PAM domain involved in ETH signaling. | [111] |
| AT2G26070 | REVERSION-TO-ETH SENSITIVITY1 (RTE1) | appears to be involved in the negative regulation of the response to ETH, is localized to the Golgi and is a positive regulator of ETR1. | [112] |
| AT4G31550 | WRKY11 | negative regulator of basal resistance | [113] |
| AT2G23320 | WRKY15 | response to chitin | [26] |
| AT4G31800 | WRKY18 | Pathogen-induced transcription factor, forms protein complexes with itself and with WRKY40 and WRKY60. WRKY18, -40, and -60 have partially redundant roles in response to the hemibiotrophic bacterial pathogen P. syringae and the necrotrophic fungal pathogen B cinerea, with WRKY18 playing a more important role than the other two. | [114 115 116] |
| AT4G01250 | WRKY22 | Functions redundantly with WRKY29 in activating early-defence genes, involved in bacterial and fungal defence responses | [87] |
| AT4G18170 | WRKY28 | Induced in response to a broad range of pathogens, confirmed co-expression with disease resistant protein encoding genes | [38] |
| AT2G38470 | WRKY33 | Regulates the antagonistic relationship between defense pathways mediating responses to P. syringae and necrotrophic fungal pathogens | [117] |
| AT1G80840 | WRKY40 | As WRKY18 | [114] |
| AT2G25000 | WRKY60 | As WRKY18 | [114 115] |
| AT3G56400 | WRKY70 | Function as activator of SA-dependent defense genes and a repressor of JA-regulated genes. WRKY70-controlled suppression of JA-signaling is partly executed by NPR1 | [118] |

## References


1. Yanhui C, et al. (2006) The MYB transcription factor superfamily of Arabidopsis: expression analysis and phylogenetic comparison with the rice MYB family. *Plant Mol Biol* 60: 107-124.

2. Lu, SX, Knowles, S M, Andronis, C, Ong, M S, Tobin, EM (2009) CIRCADIAN CLOCK ASSOCIATED1 and LATE ELONGATED HYPOCOTYL function synergistically in the circadian clock of Arabidopsis. *Plant Physiol* 150: 834-843.

3. Delaney, T (1997) Genetic dissection of acquired resistance to disease. *Plant Physiol* 113: 5-12.

4. Delaney, TP Friedrich, L, Ryals, JA (1995) Arabidopsis signal transduction mutant defective in chemically and biologically induced disease resistance. *Proc Natl Acad Sci USA* 92: 6602-6606.

5. Durrant W, Dong, X (2004) Systemic acquired resistance. *Annu Rev Phytopathol* 42: 185-209.

6. Li, J, et al. (2009) Specific ER quality control components required for biogenesis of the plant innate immune receptor EFR. *Proc Natl Acad Sci USA* 106: 15973-15978.

7. Kirik, V, et al. (2001) Ectopic expression of the Arabidopsis *AtMYB23* gene induces differentiation of trichome cells. *Dev Biol* 235: 366-377.

8. Bowling, SA, Clarke, JD, Liu, Y, Klessig, DF, Dong, X (1997) The *cpr5* mutant of Arabidopsis expresses both NPR1-dependent and NPR1-independent resistance. *Plant Cell* 9: 1573-1584.

9. Clarke, JD, Aarts, N, Feys, BJ, Dong, X, Parker, JE (2001) Constitutive disease resistance requires EDS1 in the Arabidopsis mutants *cpr1* and *cpr6* and is partially EDS1-dependent in *cpr5*. *Plant J* 26: 409-420.

10. Nawrath, C, Heck, S, Parinthawong, N, Metraux, JP (2002) EDS5, an essential component of salicylic acid-dependent signaling for disease resistance in Arabidopsis, is a member of the MATE transporter family. *Plant Cell* 14: 275-286.

11. Wildermuth, MC, Dewdney, J, Wu, G, Ausubel, FM (2001) Isochorismate synthase is required to synthesize salicylic acid for plant defence. *Nature* 414: 562-565.

12. Feys, BJ, Moisan, LJ, Newman, MA, Parker, JE (2001) Direct interaction between the Arabidopsis disease resistance signaling proteins, EDS1 and PAD4. *EMBO J* 20: 5400-5411.

13. Falk, A, et al. (1999) EDS1, an essential component of R gene-mediated disease resistance in Arabidopsis has homology to eukaryotic lipases. *Proc Natl Acad Sci USA* 96: 3292-3297.

14. Fan, W, Dong, X (2002) *In vivo* interaction between NPR1 and transcription factor TGA2 leads to salicylic acid-mediated gene activation in Arabidopsis. *Plant Cell* 14: 1377-1389.

15. Glazebrook, J,et al. (1997) Phytoalexin-Deficient mutants of Arabidopsis reveal that *Pad4* encodes a regulatory factor and that four *Pad* genes contribute to Downy Mildew resistance. *Genetics* 146: 381-392.

16. Jirage, D, et al. (1999) Arabidopsis thaliana *PAD4* encodes a lipase-like gene that is important for salicylic acid signaling. *Proc Natl Acad Sci USA* 96: 13583-13588.

17. Clay, NK, Adio, AM, Denoux, C, Jander, G, Ausubel, FM (2009) Glucosinolate metabolites required for an Arabidopsis innate immune response. *Science* 323: 95-101.

18. Glazebrook, J, Ausubel, FM (1994) Isolation of phytoalexin-deficient mutants of Arabidopsis thaliana and characterization of their interactions with bacterial pathogens. *Proc Natl Acad Sci USA* 91: 8955-8959.

19. Wanner, LA, Li, G, Ware, D, Somssich, IE, Davis, KR (1995) The phenylalanine ammonia-lyase gene family in *Arabidopsis thaliana*. *Plant Mol Biol* 27: 327-338.

20. Huang, J, et al. (2010) Functional analysis of the Arabidopsis *PAL* gene family in plant growth, development, and response to environmental stress. *Plant Physiol* 153: 1526-1538.

21. Olsen, KM, Lea, US, Slimestad, R, Verheul, M, Lillo, C (2008) Differential expression of four Arabidopsis *PAL* genes; *PAL1* and *PAL2* have functional specialization in abiotic environmental-triggered flavonoid synthesis. *J Plant Physiol* 165: 1491-1499.

22. Clough, S J, et al. (2000) The Arabidopsis *dnd1* “defense, no death” gene encodes a mutated cyclic nucleotide-gated ion channel. *Proc Natl Acad Sci USA* 97: 9323-9328.

23. Frye, CA, Tang, D, Innes, RW (2001) Negative regulation of defense responses in plants by a conserved MAPKK kinase. *Proc Natl Acad Sci USA* 98: 373-378.

24. van Hulten, M, Pelser, M, Van Loon, LC, Pieterse, CM, Ton, J (2006) Costs and benefits of priming for defense in Arabidopsis. *Proc Natl Acad Sci USA* 103: 5602-5607.

25. Gao M, et al. (2009) Regulation of cell death and innate immunity by two receptor-like kinases in Arabidopsis. *Cell Host Microbe* 6: 34-44.

26. Libault, M, Wan, J, Czechowski, T, Udvardi, M, Stacey, G (2007) Ligase genes responding to chitin, a plant-defense elicitor. *Mol Plant Microbe Interact* 20: 900-911.

27. Xiang, T, et al. (2008) *Pseudomonas syringae* effector AvrPto blocks innate immunity by targeting receptor kinases. *Curr Biol* 18: 74-80.

28. Zipfel, C, et al. (2006) Perception of the bacterial PAMP EF-Tu by the receptor EFR restricts Agrobacterium-mediated transformation. *Cell* 125: 749-760.

29. Miya, A, et al. (2007) CERK1, a LysM receptor kinase, is essential for chitin elicitor signaling in Arabidopsis. *Proc Natl Acad Sci USA* 104: 19613-19618.

30. Sanderfoot, AA, Assaad, FF, Raikhel, NV (2000) The Arabidopsis Genome. An abundance of soluble N-ethylmaleimide-sensitive factor adaptor protein receptors. *Plant Physiol* 124: 1558-1569.

31. Sanderfoot, AA, Pilgrim, M, Adam, L, Raikhel, NV (2001) Disruption of individual members of Arabidopsis syntaxin gene families indicates each has essential functions. *Plant Cell* 13: 659-666.

32. Collins, NC, et al. (2003) SNARE-protein-mediated disease resistance at the plant cell wall. *Nature* 425: 973-977.

33. Zhang, Z, et al. (2007) A SNARE-protein has opposing functions in penetration resistance and defence signalling pathways. *Plant J* 49: 302-312.

34. Lipka, V,et al. (2005) Pre- and postinvasion defenses both contribute to nonhost resistance in Arabidopsis. *Science* 310: 1180-1183.

35. Stein, M, et al. (2006) Arabidopsis PEN3/PDR8, an ATP binding cassette transporter, contributes to nonhost resistance to inappropriate pathogens that enter by direct penetration. *Plant Cell* 18: 731-746.

36. Collinge M, Boller, T (2001) Differential induction of two potato genes, *Stprx2* and *StNAC*, in response to infection by *Phytophthora infestans* and to wounding. *Plant Mol.Biol* 46: 521-529.

37. Chen, Z, et al. (2006) Expression analysis of the *AtMLO* gene family encoding plant-specific seven-transmembrane domain proteins. *Plant Mol Biol* 60: 583-597.

38. Meier S, et al. (2010) The *Arabidopsis thaliana* wall associated kinase-like protein 10 (AtWAKL10) is a dual domain enzyme with guanylyl cyclase and cGMP-dependent kinase activity *in vitro* and a role in plant defense. *PLoS ONE* 5: e8904.

39. Consonni, C, et al. (2006) Conserved requirement for a plant host cell protein in powdery mildew pathogenesis. *Nat Genet* 38: 716-720.

40. Vogel, JP, Raab, TK, Schiff, C, Somerville, SC (2002) *PMR6*, a pectate lyase-like gene required for powdery mildew susceptibility in Arabidopsis. *Plant Cell* 14: 2095-2106.

41. Vogel, JP, Raab, TK, Somerville, CR, Somerville, SC (2004) Mutations in *PMR5* result in powdery mildew resistance and altered cell wall composition. *Plant J* 40: 968-978.

42. Van Damme, M, et al. (2005) Identification of Arabidopsis loci required for susceptibility to the downy mildew pathogen *Hyaloperonospora parasitica*. *Mol Plant Microbe Interact* 18: 583-592.

43. Kwon, C et al. (2008) Co-option of a default secretory pathway for plant immune responses. *Nature* 451: 835-840.

44. Pratelli, R, Sutter, JU, Blatt, MR (2004) A new catch in the SNARE. *Trends Plant Sci* 9: 187-195.

45. Pajonk, S, Kwon, C, Clemens, N, Panstruga, R, Schulze-Lefert, P (2008) Activity determinants and functional specialization of Arabidopsis PEN1 syntaxin in innate immunity. *J Biol Chem.* 283: 26974-26984.

46. Huckelhoven, R (2007) Cell wall-associated mechanisms of disease resistance and susceptibility. *Annu Rev Phytopathol* 45: 101-127.

47. Reuber, TL, Ausubel, FM (1996) Isolation of Arabidopsis genes that differentiate between resistance responses mediated by the *RPS2* and *RPM1* disease resistance genes. *Plant Cell* 8: 241-249.

48. Bent, AF, et al. (1994) RPS2 of Arabidopsis thaliana: a leucine-rich repeat class of plant disease resistance genes. *Science* 265: 1856-1860.

49. Gopalan, S, et al. (1996) Expression of the *Pseudomonas syringae* avirulence protein AvrB in plant cells alleviates its dependence on the hypersensitive response and pathogenicity (Hrp) secretion system in eliciting genotype-specific hypersensitive cell death. *Plant Cell* 8: 1095-1105.

50. Kim, MG, et al. (2005) Two *Pseudomonas syringae* type III effectors inhibit RIN4-regulated basal defense in Arabidopsis. *Cell* 121: 749-759.

51. Wirthmueller, L, Zhang, Y, Jones, JD, Parker, JE (2007) Nuclear accumulation of the Arabidopsis immune receptor RPS4 is necessary for triggering EDS1-dependent defence. *Curr Biol* 17: 2023-2029.

52. Warren, RF, Merritt, PM, Holub, E, Innes, RW (1999) Identification of three putative signal transduction genes involved in R gene-specified disease resistance in Arabidopsis. *Genetics* 152: 401-412.

53. Feys, BJ, et al. (2005) Arabidopsis SENESCENCE-ASSOCIATED GENE101 stabilizes and signals within an ENHANCED DISEASE SUSCEPTIBILITY1 complex in plant innate immunity. *Plant Cell* 17: 2601-2613.

54. Denoux, C, et al. (2008) Activation of defense response pathways by OGs and Flg22 elicitors in Arabidopsis seedlings. *Mol.Plant* 1: 423-445.

55. Grant, JJ, Chini, A, Basu, D, Loake, GJ (2003) Targeted activation tagging of the Arabidopsis NBS-LRR gene, *ADR1*, conveys resistance to virulent pathogens. *Mol.Plant Microbe Interact.* 16, 669-680.

56. Bindschedler, LV, Palmblad, M, Cramer, R (2008) Hydroponic isotope labelling of entire plants (HILEP) for quantitative plant proteomics; an oxidative stress case study. *Phytochemistry* 69: 1962-1972.

57. Xiao, S et al. (2001) Broad-spectrum mildew resistance in *Arabidopsis thaliana* mediated by RPW8. *Science* 291: 118-120.

58. Orgil, U, Araki, H, Tangchaiburana, S, Berkey, R, Xiao, S (2007) Intraspecific genetic variations, fitness cost and benefit of *RPW8*, a disease resistance locus in *Arabidopsis thaliana*. *Genetics* 176: 2317-2333.

59. La Camera, S, et al. (2005) A pathogen-inducible patatin-like lipid acyl hydrolase facilitates fungal and bacterial host colonization in Arabidopsis. *Plant J* 44: 810-825.

60. Mukherjee, AK, et al. (2010) Proteomics of the response of *Arabidopsis thaliana* to infection with *Alternaria brassicicola*. *J Proteomics* 73: 709-720.

61. Takenaka, Y, Nakano, S, Tamoi, M, Sakuda, S, Fukamizo, T (2009) Chitinase gene expression in response to environmental stresses in *Arabidopsis thaliana*: chitinase inhibitor allosamidin enhances stress tolerance *Biosci Biotechnol Biochem* 73: 1066-1071.

62. Capelli, N, Diogon, T, Greppin, H, Simon, P (1997) Isolation and characterization of a cDNA clone encoding an osmotin-like protein from *Arabidopsis thaliana. Gene* 191: 51-56.

63. Vellosillo, T, et al. (2007) Oxylipins produced by the 9-lipoxygenase pathway in Arabidopsis regulate lateral root development and defense responses through a specific signaling cascade. *Plant Cell* 19: 831-846.

64. He, ZH, He, D, Kohorn, BD (1998) Requirement for the induced expression of a cell wall associated receptor kinase for survival during the pathogen response. *Plant J* 14: 55-63.

65. Diener AC, Ausubel, FM (2005) *RESISTANCE TO FUSARIUM OXYSPORUM 1*, a dominant Arabidopsis disease-resistance gene, is not race specific. *Genetics* 171: 305-321.

66. Pogany, M et al. (2009) Dual roles of reactive oxygen species and NADPH oxidase rbohD in an *Arabidopsis-Alternaria* pathosystem *Plant Physiol* 151: 1459-1475.

67. Torres, MA, Dangl, JL, Jones, JD (2002) Arabidopsis *gp91$^{phox}$* homologues *AtrbohD* and *AtrbohF* are required for accumulation of reactive oxygen intermediates in the plant defense response. *Proc Natl Acad Sci USA* 99: 517-522.

68. Xie, DX, Feys, BF, James, S, Nieto-Rostro, M, Turner, JG (1998) *COI1*: an Arabidopsis gene required for jasmonate-regulated defense and fertility. *Science* 280: 1091-1094.

69. Xiao, S, et al. (2004) COS1: an Arabidopsis *coronatine insensitive1* suppressor essential for regulation of jasmonate-mediated plant defense and senescence. *Plant Cell* 16: 1132-1142.

70. Coego, A, et al. (2005) An Arabidopsis homeodomain transcription factor, OVEREXPRESSOR OF CATIONIC PEROXIDASE 3, mediates resistance to infection by necrotrophic pathogens. *Plant Cell* 17: 2123-2137.

71. Staswick, PE, Tiryaki, I, Rowe, ML (2002) Jasmonate response locus *JAR1* and several related Arabidopsis genes encode enzymes of the firefly luciferase superfamily that show activity on jasmonic, salicylic, and indole-3-acetic acids in an assay for adenylation. *Plant Cell* 14: 1405-1415.

72. Laudert, D, Pfannschmidt, U, Lottspeich, F, Hollander-Czytko, H, Weiler, EW (1996) Cloning, molecular and functional characterization of Arabidopsis thaliana allene oxide synthase (CYP 74), the first enzyme of the octadecanoid pathway to jasmonates. *Plant Mol Biol* 31: 323-335.

73. Pajerowska, KM, Parker, JE, Gebhardt, C (2005) Potato homologs of *Arabidopsis thaliana* genes functional in defense signaling-identification, genetic mapping, and molecular cloning. *Mol Plant Microbe Interact.* 18: 1107-1119.

74. Sanders, PM, et al. (2000) The Arabidopsis *DELAYED DEHISCENCE1* gene encodes an enzyme in the jasmonic acid synthesis pathway. *Plant Cell* 12: 1041-1062.

75. Schilmiller, AL, Koo, AJ, Howe, GA (2007) Functional diversification of acyl-coenzyme A oxidases in jasmonic acid biosynthesis and action. *Plant Physiol* 143: 812-824.

76. Seo, HS, et al. (2001) Jasmonic acid carboxyl methyltransferase: a key enzyme for jasmonate-regulated plant responses. *Proc Natl Acad Sci USA* 98: 4788-4793.

77. Kariola, T, Brader, G, Li, J, Palva, ET (2005) Chlorophyllase 1, a damage control enzyme, affects the balance between defense pathways in plants. *Plant Cell* 17: 282-294.

78. Leon, J, Rojo, E, Titarenko, E, Sanchez-Serrano, JJ (1998) Jasmonic acid-dependent and -independent wound signal transduction pathways are differentially regulated by $Ca^{2+}$/calmodulin in *Arabidopsis thaliana*. *Mol Gen Genet* 258: 412-419.

79. Lopukhina, A, Dettenberg, M, Weiler, EW, Hollander-Czytko, H (2001) Cloning and characterization of a coronatine-regulated tyrosine aminotransferase from Arabidopsis. *Plant Physiol* 126: 1678-1687.

80. Sels, J, Mathys, J, De Coninck, BM, Cammue, BP, De Bolle, MF (2008) Plant pathogenesis-related (PR) proteins: a focus on PR peptides. *Plant Physiol Biochem* 46: 941-950.

81. Lorenzo, O, Chico, JM, Sanchez-Serrano, JJ, Solano, R (2004) *JASMONATE-INSENSITIVE1* encodes a MYC transcription factor essential to discriminate between different jasmonate-regulated defense responses in Arabidopsis. *Plant Cell* 16: 1938-1950.

82. Dombrecht, B, et al. (2007) MYC2 differentially modulates diverse jasmonate-dependent functions in Arabidopsis. *Plant Cell* 19: 2225-2245.

83. Bohlmann, H, et al. (1998) Wounding and chemicals induce expression of the *Arabidopsis thaliana* gene *Thi2. 1*, encoding a fungal defense thionin, via the octadecanoid pathway. *FEBS Lett* 437: 281-286.

84. Pye, VE, Christensen, CE, Dyer, JH, Arent, S, Henriksen, A (2010) Peroxisomal plant 3-ketoacyl-CoA thiolase structure and activity are regulated by a sensitive redox switch. *J Biol Chem.* 285: 24078-24088.

85. Carrie, C, Murcha, MW, Millar, AH, Smith, SM, Whelan, J (2007) Nine 3-ketoacyl-CoA thiolases (KATs) and acetoacetyl-CoA thiolases (ACATs) encoded by five genes in *Arabidopsis thaliana* are targeted either to peroxisomes or cytosol but not to mitochondria. *Plant Mol Biol* 63: 97-108.

86. Austin, MJ, et al. (2002) Regulatory role of SGT1 in early R gene-mediated plant defenses. *Science* 295: 2077-2080.

87. Tor, M, et al. (2002) Arabidopsis *SGT1b* is required for defense signaling conferred by several downy mildew resistance genes. *Plant Cell* 14: 993-1003.

88. Maldonado, A, Doerner, P, Dixon, R, Lamb, C, Cameron, R (2002) A putative lipid transfer protein involved in systemic resistance signalling in Arabidopsis. *Nature* 419: 399-403.

89. Asai, T, et al. (2002) MAP kinase signalling cascade in Arabidopsis innate immunity. *Nature* 415: 977-983.

90. Doczi R, et al. (2007) The Arabidopsis mitogen-activated protein kinase kinase MKK3 is upstream of group C mitogen-activated protein kinases and participates in pathogen signaling. *Plant Cell* 19: 3266-3279.

91. Brodersen, P, et al. (2006) Arabidopsis MAP kinase 4 regulates salicylic acid-and jasmonic acid/ethylene-dependent responses via EDS1 and PAD4. *Plant J* 47: 532-546.

92. Beckers, GJ, et al. (2009) Mitogen-activated protein kinases 3 and 6 are required for full priming of stress responses in *Arabidopsis thaliana*. *Plant Cell* 21: 944-953.

93. Qiu, JL, et al. (2008) Arabidopsis mitogen-activated protein kinase kinases MKK1 and MKK2 have overlapping functions in defense signaling mediated by MEKK1, MPK4, and MKS1. *Plant Physiol* 148: 212-222.

94. Xu, J, et al. (2008) Activation of MAPK kinase 9 induces ethylene and camalexin biosynthesis and enhances sensitivity to salt stress in Arabidopsis. *J Biol Chem* 283: 26996-27006.

95. Lee, JS, Ellis, BE (2007) Arabidopsis MAPK phosphatase 2 (MKP2) positively regulates oxidative stress tolerance and inactivates the MPK3 and MPK6 MAPKs. *J Biol Chem* 282: 25020-25029.

96. Lampard, GR, Macalister, CA, Bergmann, DC (2008) Arabidopsis stomatal initiation is controlled by MAPK-mediated regulation of the bHLH SPEECHLESS. *Science* 322: 1113-1116.

97. Ortiz-Masia, D, Perez-Amador, MA, Carbonell, J, Marcote, MJ (2007) Diverse stress signals activate the C1 subgroup MAP kinases of Arabidopsis. *FEBS Lett* 581: 1834-1840.

98. Zhang, X, et al. (2007) Overexpression of Arabidopsis *MAP kinase kinase 7* leads to activation of plant basal and systemic acquired resistance. *Plant J* 52: 1066-1079.

99. Hadiarto, T et al. (2006) Activation of Arabidopsis MAPK kinase kinase (AtMEKK1) and induction of AtMEKK1–AtMEK1 pathway by wounding. *Planta* 223: 708-713.

100. Chini, A, et al. (2007) The JAZ family of repressors is the missing link in jasmonate signalling. *Nature* 448: 666-671.

101. Melotto, M, et al. (2008) A critical role of two positively charged amino acids in the Jas motif of Arabidopsis JAZ proteins in mediating coronatine- and jasmonoyl isoleucine-dependent interactions with the COI1 F-box protein. *Plant J* 55: 979-988.

102. Thines, B, et al. (2007) JAZ repressor proteins are targets of the SCFCOI1 complex during jasmonate signalling. *Nature* 448: 661-665.

103. Yan, Y, et al. (2007) A downstream mediator in the growth repression limb of the jasmonate pathway. *Plant Cell* 19: 2470-2483.

104. Pauwels, L, et al. (2010) NINJA connects the co-repressor TOPLESS to jasmonate signalling. *Nature* 464: 788-791.

105. Truman, WM, Bennett, MH, Turnbull, CG, Grant, MR (2010) Arabidopsis auxin mutants are compromised in systemic acquired resistance and exhibit aberrant accumulation of various indolic compounds. *Plant Physiol* 152: 1562-1573.

106. Lorenzo, O, Piqueras, R, Sanchez-Serrano, JJ, Solano, R (2003) ETHYLENE RESPONSE FACTOR1 integrates signals from ethylene and jasmonate pathways in plant defense. *Plant Cell* 15: 165-178.

107. Kang, HG, Fang, Y, Singh, KB (1999) A glucocorticoid-inducible transcription system causes severe growth defects in Arabidopsis and induces defense-related genes. *Plant J* 20: 127-133.

108. McGrath, KC, et al. (2005) Repressor- and activator-type ethylene response factors functioning in jasmonate signaling and disease resistance identified via a genome-wide screen of Arabidopsis transcription factor gene expression. *Plant Physiol* 139: 949-959.

109. Adie, BA, et al. (2007) ABA is an essential signal for plant resistance to pathogens affecting JA biosynthesis and the activation of defenses in Arabidopsis. *Plant Cell* 19: 1665-1681.

110. Chang, C, Kwok, SF, Bleecker, AB, Meyerowitz, EM (1993) Arabidopsis ethylene-response gene *ETR1*: similarity of product to two-component regulators. *Science* 262: 539-544.

111. Christians, MJ, Robles, LM, Zeller, SM, Larsen, PB (2008) The *eer5* mutation, which affects a novel proteasome-related subunit, indicates a prominent role for the COP9 signalosome in resetting the ethylene-signaling pathway in Arabidopsis. *Plant J* 55: 467-477.

112. Resnick, JS, Wen, CK, Shockey, JA, Chang, C (2006) *REVERSION-TO-ETHYLENE SENSITIVITY1*, a conserved gene that regulates ethylene receptor function in Arabidopsis. *Proc Natl Acad Sci USA* 103: 7917-7922.

113. Journot-Catalino, N, Somssich, IE, Roby, D, Kroj, T (2006) The transcription factors WRKY11 and WRKY17 act as negative regulators of basal resistance in *Arabidopsis thaliana*. *Plant Cell* 18: 3289-3302.

114. Chen CH, Chen, ZX (2002) Potentiation of developmentally regulated plant defense response by AtWRKY18, a pathogen-induced Arabidopsis transcription factor. *Plant Physiol* 129: 706-716.

115. Xu, X, Chen, C, Fan, B, Chen, Z (2006) Physical and functional interactions between pathogen-induced Arabidopsis WRKY18, WRKY40, and WRKY60 transcription factors. *Plant Cell* 18: 1310-1326.

116. Dong, J, Chen, C, Chen, Z (2003) Expression profiles of the Arabidopsis *WRKY* gene superfamily during plant defense response. *Plant Mol Biol* 51: 21-37.

117. Zheng, Z, Qamar, SA, Chen, Z, Mengiste, T (2006) Arabidopsis WRKY33 transcription factor is required for resistance to necrotrophic fungal pathogens. *Plant J* 48: 592-605.

118. Li, J, Brader, G, Kariola, T, Palva, ET (2006) WRKY70 modulates the selection of signaling pathways in plant defense. *Plant J* 46: 477-491.
