# Supplementary material for: Defence Responses of Arabidopsis thaliana to Infection by Pseudomonas syringae Are Regulated by the Circadian Clock
Source: PLoS One. 2011 Oct 31;6(10):e26968. doi: 10.1371/journal.pone.0026968 (PMC3205005; doi:10.1371/journal.pone.0026968)
Supplement: Table S2 — Genes exhibiting diurnal expression pattern. Genes highlighted in blue also display circadian patterns of expression. Known circadian genes CCA1 and LHY are highlighted in red. Genes determined to have diurnal expression by both Multi Experiment Viewer clustering and HAYSTACK are indicated in bold. The cluster into which each gene falls (in Figure S1) and the phase of transcript accumulation according to HAYSTACK is indicated. (PDF) [file pone.0026968.s005.pdf]

Table S2: Genes exhibiting diurnal expression pattern. Genes highlighted in blue also display circadian patterns of expression. Known circadian genes *CCA1* and *LHY* are highlighted in red. Genes determined to have circadian expression by both Multi Experiment Viewer clustering and HAYSTACK are indicated in bold. The cluster into which each gene falls (in Figure S1) and the phase of transcript accumulation according to HAYSTACK is indicated.

Probe ID	Locus	Cluster	phase
**256243_at**	**At3g12500_BASIC CHITINASE (CHI-B); (B-CHI)**	**1**	**CT22**
248895_at	AT5G46330_FLAGELLIN-SENSITIVE 2 (FLS2)	1	
248719_at	AT5G47910_RESPIRATORY BURST OXIDASE HOMOLOGUE D (RBOHD)	1	
**248247_at**	**AT5G53210_SPEECHLESS (SPCH)**	**1**	**CT03**
256425_at	AT1G33560_ACTIVATED DISEASE RESISTANCE 1 (ADR1)	2	
**260116_at**	**AT1G33960_AVRRPT2-INDUCED GENE 1 (AIG1)**	**2**	**CT00**
260133_at	AT1G66340_ETHYLENE RESPONSE 1 (ETR1)	2	
262177_at	AT1G74710_ISOCHORISMATE SYNTHASE 1 (ICS1)	2	
266893_at	AT2G26070_REVERSION-TO-ETHYLENE SENSITIVITY1 (RTE1)	2	
**254652_at**	**AT4G18170_(AtWRKY28)**	**2**	**CT03**
245690_at	AT5G04230_PHENYL ALANINE AMMONIA-LYASE 3 (PAL3)	2	
261569_at	AT1G01060_LATE ELONGATED HYPOCOTYL (LHY)	5	CT23
**261564_at**	**AT1G01720_ (ATAF1)**	5	
264780_at	AT1G08720_ENHANCED DISEASE RESISTANCE 1 (EDR1); (ATEDR1)	5	
**261135_at**	**At1g19610_ (PDF1.4)**	**5**	**CT21**
**261150_at**	**AT1G19640_JASMONIC ACID CARBOXYL METHYLTRANSFERASE (JMT)**	**5**	**CT22**
259802_at	AT1G72260_THIONIN 2.1 (THI2.1); (THI2.1.1)	5	
261402_at	AT1G79670_RESISTANCE TO FUSARIUM OXYSPORUM 1 (RFO1); (WAKL22)	5	
**266141_at**	**At2g02120_ (PDF2.1)**	**5**	**CT00**
265934_at	AT2G19560_ENHANCED ETHYLENE RESPONSE 5 (EER5)	5	
**245051_at**	**AT2G23320_(WRKY15)**	**5**	**CT22**
**257365_at**	**At2g26020_plant defensin 1.2b (PDF1.2b)**	**5**	**CT01**
266992_at	AT2G39200_MILDEW RESISTANCE LOCUS O 12 (MLO12)	5	
267346_at	AT2G39940_CORONATINE INSENSITIVE 1 (COI1)	5	
260571_at	AT2G43790_MAP KINASE 6 (MPK6);MAP KINASE 6 (MAPK6)	5	
266719_at	AT2G46830_CIRCADIAN CLOCK ASSOCIATED 1 (CCA1)	5	CT00
256397_at	AT3G06110_MAPK PHOSPHATASE 2 (MKP2)	5	
**259149_at**	**AT3G10340_Phenylalanine ammonia-lyase 4 (PAL4)**	**5**	**CT23**
**258434_at**	**AT3G16770_;ETHYLENE RESPONSE FACTOR 72 (ERF72)**	**5**	**CT01**
258189_at	AT3G17860_JASMONATE-ZIM-DOMAIN PROTEIN 3 (JAZ3)	5	
**251984_at**	**AT3G53260_PHENYLALANINE AMMONIA-LYASE 2 (PAL2)**	**5**	**CT23**
**251864_at**	**AT3G54920_powdery mildew resistant 6 (PMR6)**	**5**	**CT21**
**255568_at**	**AT4G01250_ (WRKY22); (AtWRKY22)**	**5**	**CT21**
245249_at	AT4G16760_ACYL-COA OXIDASE 1 (ACX1); (ATACX1)	5	
250421_at	AT5G11270_OVEREXPRESSOR OF CATIONIC PEROXIDASE 3 (OCP3)	5	
246510_at	AT5G15410_DEFENSE NO DEATH 1 (DND1)	5	
**248994_at**	**AT5G45250_RESISTANT TO P. SYRINGAE 4 (RPS4)**	**5**	**CT21**

**248684_at**	**AT5G48485_DEFECTIVE IN INDUCED RESISTANCE 1 (DIR1)**	**5**	**CT22**
**247259_at**	**AT5G64930_CONSTITUTIVE EXPRESSION OF PR GENES 5 (CPR5)**	**5**	**CT23**
245986_at	AT5G13160_avrPphB susceptible 1 (PBS1)	5	
**256017_at**	**AT1G19180_JASMONATE-ZIM-DOMAIN PROTEIN 1 (JAZ1);**	**6**	**CT07**
**261713_at**	**AT1G32640_JASMONATE INSENSITIVE 1 (JIN1); (MYC2))**	**6**	**CT10**
**262899_at**	**AT1G59870_PENETRATION 3 (PEN3)**	**6**	**CT10**
**265530_at**	**AT2G06050_OPDA-REDUCTASE 3 (OPR3)**	**6**	**CT10**
**267392_at**	**AT2G44490_penetration 2 (PEN2);BETA GLUCOSIDASE 26 (BGLU26)**	**6**	**CT10**
263786_at	AT2G46370_JASMONATE RESISTANT 1 (JAR1)	6	
258791_at	At3g04720_PATHOGENESIS-RELATED 4 (PR4)	6	
258046_at	AT3G21220_ (ATMKK5)	6	
258173_at	AT3G21630_Chitin Elicitor Receptor Kinase 1 (CERK1)	6	
252373_at	AT3G48090_enhanced disease susceptibility 1 (EDS1)	6	
**254232_at**	**AT4G23600_CORONATINE INDUCED 1 (CORI3)**	**6**	**CT10**
**246082_at**	**AT5G20480_EF-TU RECEPTOR (EFR)**	**6**	**CT09**
249754_at	AT5G24530_DOWNY MILDEW RESISTANT 6 (DMR6)	6	
**249560_at**	**At5g38330_Low-molecular-weight cysteine-rich 80 (LCR80)**	**6**	**CT07**
**249208_at**	**AT5G42650_ALLENE OXIDE SYNTHASE (AOS)**	**6**	**CT10**
249052_at	At5g44420_PLANT DEFENSIN 1.2 (PDF1.2)	6	
264595_at	AT1G04750_VESICLE-ASSOCIATED MEMBRANE PROTEIN 721	7	
**262455_at**	**AT1G11310_MILDEW RESISTANCE LOCUS O 2 (MLO2)**	7	
**261662_at**	**AT1G18350_MAP KINASE KINASE7 (ATMKK7)**	**7**	**CT11**
255786_at	AT1G19670_CORONATINE-INDUCED PROTEIN 1 (ATCLH1)	7	
**259561_at**	**AT1G21250_CELL WALL-ASSOCIATED KINASE (WAK1); (PRO25)**	**7**	**CT10**
256183_at	AT1G51660_(MKK4)	7	
265008_at	AT1G61560_MILDEW RESISTANCE LOCUS O 6 (MLO6)	7	
259764_at	AT1G64280_NONEXPRESSER OF PR GENES 1 (NPR1)	7	
**259925_at**	**AT1G75040_PATHOGENESIS-RELATED GENE 5 (PR5); (PR-5)**	**7**	**CT11**
**262679_at**	**At1g75830_PLANT DEFENSIN 1.2 (PDF1.1)**	**7**	**CT12**
**266119_at**	**At2g02100_(PDF2.2)**	**7**	**CT10**
266118_at	At2g02130_ (PDF2.3)	7	
**245038_at**	**At2g26560_PHOSPHOLIPASE A 2A (PLP2)**	**7**	**CT10**
263478_at	AT2G31880_SUPPRESSOR OF BIR1 1 (SOBIR1)	7	
245168_at	AT2G33150_(KAT2)	7	
258786_at	AT3G11820_PENETRATION1 (PEN1)	7	
257053_at	At3g15210_ETHYLENE RESPONSIVE ELEMENT BINDING FACTOR 4 (ATERF-4)	7	
**251705_at**	**AT3G56400_ (WRKY70)**	**7**	**CT10**
255624_at	AT4G01370_MPK4	7	
**253993_at**	**AT4G26070_ (MKK1)**	**7**	**CT10**
**253997_at**	**AT4G26090_RESISTANT TO P. SYRINGAE 2 (RPS2)**	**7**	**CT10**
**247786_at**	**AT5G58600_POWDERY MILDEW RESISTANT 5 (PMR5)**	**7**	**CT13**
**247337_at**	**At5g63660_(PDF2.5)**	**7**	**CT13**
**259451_at**	**AT1G13890_ (SNAP30)**	**9**	
245731_at	AT1G73500_MAP KINASE KINASE 9 (MKK9)	9	
**257927_at**	**AT3G23240_ETHYLENE RESPONSE FACTOR 1 (ERF1)**	**9**	**CT16**

**258002_at**	**At3g28930_AVRRPT2-INDUCED GENE 2 (AIG2)**	**9**	**CT13**
**254889_at**	**At4g11650_osmotin 34 (ATOSM34)**	**9**	**CT16**
252921_at	AT4G39030_ENHANCED DISEASE SUSCEPTIBILITY 5 (EDS5)	9	
**250551_at**	**AT5G07880_(SNAP29)**	**9**	**CT11**
**249645_at**	**At5g36910_THIONIN 2.2 (THI2.2)**	**9**	**CT10**
262344_at	AT1G64060_RESPIRATORY BURST OXIDASE PROTEIN F (ATRBOH F)	10	
267028_at	AT2G38470_ (WRKY33)	10	
252592_at	AT3G45640_(ATMAPK3)	10	
255378_at	AT4G03550_GLUCAN SYNTHASE-LIKE 5 (ATGSL05)	10	
**253646_at**	**AT4G29810_MAP KINASE KINASE 2 (MKK2);**	**10**	**CT11**
**266385_at**	**AT2G14610_PATHOGENESIS-RELATED GENE 1 (PR1)**	**10**	**CT08**
254211_at	AT4G23570_ (SGT1A)	10	
**253535_at**	**AT4G31550_ (AtWRKY11)**	**10**	**CT09**
259719_at	At1g61070_PLANT DEFENSIN 2.4 (PDF2.4)		CT12
266115_at	At2g02140_ (PDF2.6)		CT20
252170_at	AT3G50480_HOMOLOG OF RPW8 4 (HR4)		CT17
252060_at	AT3G52430_PHYTOALEXIN DEFICIENT 4 (PAD4)		CT17
251625_at	AT3G57260_PATHOGENESIS-RELATED PROTEIN 2 (PR2)		CT11
