# Supplementary material for: Defence Responses of Arabidopsis thaliana to Infection by Pseudomonas syringae Are Regulated by the Circadian Clock
Source: PLoS One. 2011 Oct 31;6(10):e26968. doi: 10.1371/journal.pone.0026968 (PMC3205005; doi:10.1371/journal.pone.0026968)
Supplement: Table S3 — Genes exhibiting circadian expression pattern. Genes highlighted in yellow also display diurnal patterns of expression. Known circadian genes CCA1 and LHY are highlighted in red. Genes determined to have circadian expression by both Multi Experiment Viewer clustering and HAYSTACK are indicated in bold. The cluster into which each gene falls (in Figure S2) and the phase of transcript accumulation according to HAYSTACK is indicated. (PDF) [file pone.0026968.s006.pdf]

Table S3: Genes exhibiting circadian expression pattern. Genes highlighted in yellow also display diurnal patterns of expression. Known circadian genes *CCA1* and *LHY* are highlighted in red. Genes determined to have circadian expression by both Multi Experiment Viewer clustering and HAYSTACK are indicated in bold. The cluster into which each gene falls (in Figure S2) and the phase of transcript accumulation according to HAYSTACK is indicated.

Probe ID	Locus	Cluster	phase
261135_at	At1g19610_ (PDF1.4)	1	
261394_at	AT1G79680_wakl10	1	
**266115_at**	**At2g02140_ (PDF2.6)**	**1**	**CT03**
266385_at	AT2G14610_PATHOGENESIS-RELATED GENE 1 (PR1)	1	
266893_at	AT2G26070_REVERSION-TO-ETHYLENE SENSITIVITY1 (RTE1)	1	
267028_at	AT2G38470_ (WRKY33)	1	
266992_at	AT2G39200_MILDEW RESISTANCE LOCUS O 12 (MLO12)	1	
260571_at	AT2G43790_MAP KINASE 6 (MPK6);MAP KINASE 6 (MAPK6)	1	
258189_at	AT3G17860_JASMONATE-ZIM-DOMAIN PROTEIN 3 (JAZ3)	1	
258046_at	AT3G21220_ (ATMKK5)	1	
257868_at	AT3G25070_RPM1 INTERACTING PROTEIN 4 (RIN4)	1	
**252373_at**	**AT3G48090_enhanced disease susceptibility 1 (EDS1)**	**1**	**CT04**
250928_at	AT5G03280_ETHYLENE INSENSITIVE 2 (EIN2)	1	
**245690_at**	**AT5G04230_PHENYL ALANINE AMMONIA-LYASE 3 (PAL3)**	**1**	**CT04**
**246082_at**	**AT5G20480_EF-TU RECEPTOR (EFR)**	**1**	**CT03**
**249645_at**	**At5g36910_THIONIN 2.2 (THI2.2)**	**1**	**CT03**
249754_at	AT5G24530_DOWNY MILDEW RESISTANT 6 (DMR6)	1	
264595_at	AT1G04750_VESICLE-ASSOCIATED MEMBRANE PROTEIN 721	2	
261892_at	AT1G80840_ (ATWRKY40)	2	
245168_at	AT2G33150_(KAT2)	2	
267392_at	AT2G44490_penetration 2 (PEN2);BETA GLUCOSIDASE 26 (BGLU26)	2	
**258791_at**	**At3g04720_PATHOGENESIS-RELATED 4 (PR4)**	**2**	**CT07**
258786_at	AT3G11820_PENETRATION1 (PEN1)	2	
252592_at	AT3G45640_(ATMAPK3)	2	
254918_at	AT4G11260_ENHANCED DOWNY MILDEW 1 (EDM1); (SGT1B)	2	
**254889_at**	**At4g11650_osmotin 34 (ATOSM34)**	**2**	**CT08**
253993_at	AT4G26070_ (MKK1)	2	
253377_at	AT4G33300_ADR1-like 1 (ADR1-L1)	2	
246600_at	AT5G14930_SENESCENCE-ASSOCIATED GENE 101	2	
**249560_at**	**At5g38330_Low-molecular-weight cysteine-rich 80 (LCR80)**	**2**	**CT08**
**248895_at**	**AT5G46330_FLAGELLIN-SENSITIVE 2 (FLS2)**	**2**	**CT07**
**247571_at**	**AT5G61210_ (SNAP33)**	**2**	**CT07**
262455_at	AT1G11310_MILDEW RESISTANCE LOCUS O 2 (MLO2)	4	
262344_at	AT1G64060_RESPIRATORY BURST OXIDASE PROTEIN F (ATRBOH F)	4	
266119_at	At2g02100_(PDF2.2)	4	
258173_at	AT3G21630_Chitin Elicitor Receptor Kinase 1 (CERK1)	4	

250421_at	AT5G11270_OVEREXPRESSOR OF CATIONIC PEROXIDASE 3 (OCP3)	4	
248994_at	AT5G45250_RESISTANT TO P. SYRINGAE 4 (RPS4)	4	
**248247_at**	**AT5G53210_SPEECHLESS (SPCH)**	**4**	**CT09**
**247786_at**	**AT5G58600_POWDERY MILDEW RESISTANT 5 (PMR5)**	**4**	**CT10**
**264405_at**	**AT1G10210_(ATMPK1)**	**5**	**CT07**
**261662_at**	**AT1G18350_MAP KINASE KINASE7 (ATMKK7)**	**5**	**CT20**
**262899_at**	**AT1G59870_PENETRATION 3 (PEN3)**	**5**	**CT09**
263786_at	AT2G46370_JASMONATE RESISTANT 1 (JAR1)	5	
**258544_at**	**AT3G07040_(RPM1)**	**5**	**CT11**
259149_at	AT3G10340_Phenylalanine ammonia-lyase 4 (PAL4)	5	
**256243_at**	**At3g12500_BASIC CHITINASE (CHI-B); (B-CHI)**	**5**	**CT06**
**258434_at**	**AT3G16770_;ETHYLENE RESPONSE FACTOR 72 (ERF72)**	**5**	**CT10**
**255568_at**	**AT4G01250_ (WRKY22); (AtWRKY22)**	**5**	**CT05**
253535_at	AT4G31550_ (AtWRKY11)	5	
**249052_at**	**At5g44420_PLANT DEFENSIN 1.2 (PDF1.2)**	**5**	**CT12**
264780_at	AT1G08720_ENHANCED DISEASE RESISTANCE 1 (EDR1); (ATEDR1)	5	
**261150_at**	**AT1G19640_JASMONIC ACID CARBOXYL METHYLTRANSFERASE (JMT)**	**10**	**CT20**
**259719_at**	**At1g61070_PLANT DEFENSIN 2.4 (PDF2.4)**	**10**	**CT21**
**245051_at**	**AT2G23320_(WRKY15)**	**10**	**CT19**
267188_at	AT2G44050_COI1 SUPPRESSOR1 (COS1)	10	
258002_at	At3g28930_AVRRPT2-INDUCED GENE 2 (AIG2)	10	
245986_at	AT5G13160_avrPphB susceptible 1 (PBS1)	10	
**248684_at**	**AT5G48485_DEFECTIVE IN INDUCED RESISTANCE 1 (DIR1)**	**10**	**CT18**
256183_at	AT1G51660_(MKK4)	10	
261569_at	AT1G01060_LATE ELONGATED HYPOCOTYL (LHY)	11	
**262177_at**	**AT1G74710_ISOCHORISMATE SYNTHASE 1 (ICS1)**	**11**	**CT23**
262679_at	At1g75830_PLANT DEFENSIN 1.2 (PDF1.1)	11	
**266118_at**	**At2g02130_ (PDF2.3)**	**11**	**CT00**
267346_at	AT2G39940_CORONATINE INSENSITIVE 1 (COI1)	11	
266719_at	AT2G46830_CIRCADIAN CLOCK ASSOCIATED 1 (CCA1)	11	
**251984_at**	**AT3G53260_PHENYLALANINE AMMONIA-LYASE 2 (PAL2)**	**11**	**CT22**
246510_at	AT5G15410_DEFENSE NO DEATH 1 (DND1)	11	
247259_at	AT5G64930_CONSTITUTIVE EXPRESSION OF PR GENES 5 (CPR5)	11	
252921_at	AT4G39030_ENHANCED DISEASE SUSCEPTIBILITY 5 (EDS5)	11	
259451_at	AT1G13890_ (SNAP30)		CT00
256017_at	AT1G19180_JASMONATE-ZIM-DOMAIN PROTEIN 1 (JAZ1)		CT00
245731_at	AT1G73500_MAP KINASE KINASE 9 (MKK9)		CT00
260060_at	At1g73680_ALPHA DIOXYGENASE (ALPHA DOX2)		CT00
260116_at	AT1G33960_AVRRPT2-INDUCED GENE 1 (AIG1)		CT01
257053_at	At3g15210_ETHYLENE RESPONSIVE ELEMENT BINDING FACTOR 4 (ATERF-4)		CT01
245038_at	At2g26560_PHOSPHOLIPASE A 2A (PLP2)		CT03
253997_at	AT4G26090_RESISTANT TO P. SYRINGAE 2 (RPS2)		CT03
266992_at	AT2G39200_MILDEW RESISTANCE LOCUS O 12 (MLO12)		CT03
251864_at	AT3G54920_powdery mildew resistant 6 (PMR6)		CT08
261713_at	AT1G32640_JASMONATE INSENSITIVE 1 (JIN1); (MYC2))		CT11

257927_at	AT3G23240_ETHYLENE RESPONSE FACTOR 1 (ERF1)	CT15
265530_at	AT2G06050_OPDA-REDUCTASE 3 (OPR3)	CT15
253485_at	AT4G31800_(WRKY18)	CT16
264608_at	AT1G04710_3-KETO-ACYL-COA THIOLASE 1 (KAT1)	CT16
266141_at	At2g02120_ (PDF2.1)	CT18
261564_at	AT1G01720_ (ATAF1)	CT18
254232_at	AT4G23600_CORONATINE INDUCED 1 (CORI3)	CT18
249208_at	AT5G42650_ALLENE OXIDE SYNTHASE (AOS)	CT18
255786_at	AT1G19670_CORONATINE-INDUCED PROTEIN 1 (ATCLH1)	CT20
257365_at	At2g26020_plant defensin 1.2b (PDF1.2b)	CT20
252060_at	AT3G52430_PHYTOALEXIN DEFICIENT 4 (PAD4)	CT20
254652_at	AT4G18170_(AtWRKY28)	CT21
251705_at	AT3G56400_ (WRKY70)	CT23
259561_at	AT1G21250_CELL WALL-ASSOCIATED KINASE (WAK1); (PRO25)	CT23
